# Overexpression of Cx43 in cells of the myocardial scar: Correction of post-infarct arrhythmias through heterotypic cell-cell coupling

**DOI:** 10.1038/s41598-018-25147-8

**Published:** 2018-05-08

**Authors:** Wilhelm Roell, Alexandra M. Klein, Martin Breitbach, Torsten S. Becker, Ashish Parikh, Jane Lee, Katrin Zimmermann, Shaun Reining, Beth Gabris, Annika Ottersbach, Robert Doran, Britta Engelbrecht, Miriam Schiffer, Kenichi Kimura, Patricia Freitag, Esther Carls, Caroline Geisen, Georg D. Duerr, Philipp Sasse, Armin Welz, Alexander Pfeifer, Guy Salama, Michael Kotlikoff, Bernd K. Fleischmann

**Affiliations:** 1 Department of Cardiac Surgery, University of Bonn, Sigmund Freud Str. 25, 53127 Bonn, Germany; 2 Institute of Physiology I, Life&Brain Center, Medical Faculty, University of Bonn, Sigmund Freud Str. 25, 53127 Bonn, Germany; 3 Department of Biomedical Sciences, College of Veterinary Medicine, Cornell University, T4-018 Veterinary Research Tower, 14853-2703 Ithaca, NY USA; 4 Department of Medicine, Heart and Vascular Institute and the McGowan Institute for Regenerative Medicine, University of Pittsburgh, School of Medicine, 3500 Terrace Street, S368 Scaife Hall, 15261 Pittsburgh, PA USA; 5 Department of Pharmacology and Toxicology, Biomedical Center, University of Bonn, Sigmund Freud Str. 25, 53127 Bonn, Germany

## Abstract

Ventricular tachycardia (VT) is the most common and potentially lethal complication following myocardial infarction (MI). Biological correction of the conduction inhomogeneity that underlies re-entry could be a major advance in infarction therapy. As minimal increases in conduction of infarcted tissue markedly influence VT susceptibility, we reasoned that enhanced propagation of the electrical signal between non-excitable cells within a resolving infarct might comprise a simple means to decrease post-infarction arrhythmia risk. We therefore tested lentivirus-mediated delivery of the gap-junction protein Connexin 43 (Cx43) into acute myocardial lesions. Cx43 was expressed in (myo)fibroblasts and CD45^+^ cells within the scar and provided prominent and long lasting arrhythmia protection *in vivo*. Optical mapping of Cx43 injected hearts revealed enhanced conduction velocity within the scar, indicating Cx43-mediated electrical coupling between myocytes and (myo)fibroblasts. Thus, Cx43 gene therapy, by direct *in vivo* transduction of non-cardiomyocytes, comprises a simple and clinically applicable biological therapy that markedly reduces post-infarction VT.

## Introduction

Ventricular tachycardia (VT) is the most common and potentially lethal complication following myocardial infarction (MI)^1^. Current experimental strategies for treatment include the generation of new myocytes within the infarct through fibroblast re-programming^2^, cell cycle re-activation in quiescent heart muscle cells^3^ or transplantation of *in-vitro* differentiated pluripotent cells^4,5^. Each of these approaches presents significant challenges, however, and their use as effective therapies in humans is likely many years away. Non-biological treatments, particularly the broad application of implanted cardiac defibrillators (ICD), have led to a marked decrease of mortality rates^6,7^, however there remain significant limitations to ICD approaches to managing post-infarction arrhythmia^8,9^. Therefore, a simple biological correction of the conduction inhomogeneity between normal and infarcted myocardium that underlies re-entry^10,11^ would comprise a major advance in infarction therapy, addressing one of the major causes of lethality in otherwise healthy individuals.

As the gap junction protein Connexin 43 (Cx43) mediates electrical coupling between ventricular cardiomyocytes^12,13^ and engraftment of Cx43 expressing embryonic cardiomyocytes or transgenic skeletal myoblasts strongly reduces VT incidence following MI^14^, we reasoned that expression of Cx43 in the connective tissue, inflammatory cells, and vascular elements that replace myocytes within the scarred myocardium could enhance conduction and reduce the incidence of post-infarct VT *in vivo*. To explore this hypothesis, we overexpressed Cx43 shortly after the infarction in resident cells within the scar using a stably integrating lentivirus^15^ as a simple means to achieve a long lasting protection against post-infarction arrhythmia. The lentivirus-mediated transduction strategy was chosen, because we anticipated that this could provide reliable overexpression of Cx43 in non-excitable cells of the scar, in particular of myofibroblasts. Here we demonstrate that this approach is an effective biological strategy to enhance heterotypic cell-cell electrical coupling and reduce VT incidence *in vivo*.

## Results

### Lentiviral-based overexpression of Cx43 generates functional gap junctions

**Figure 1:**
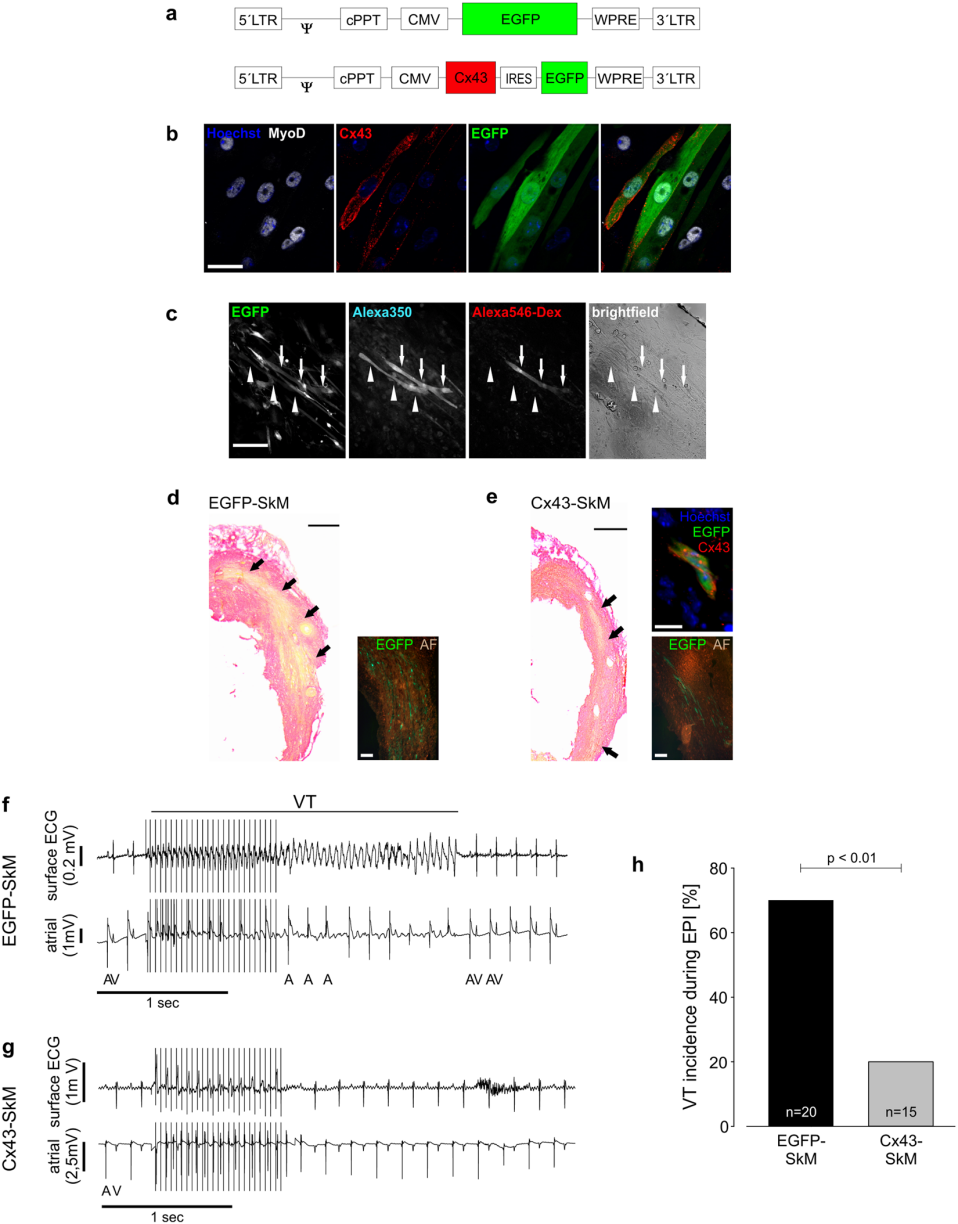
Lentivirus-mediated Cx43 transduction of SkM results in functional gap junction formation *in vitro* and *in vivo*. (**a**) Scheme of the control EGFP and the bicistronic Cx43 lentiviral vectors. (**b**) Immunostainings of cultured, lentivirus transduced EGFP^+^ (green, third panel from left; nuclear Hoechst stain, blue) SkM prove expression of MyoD (white, left panel) and membrane-located Cx43 (red, second panel from left); the right panel is an overlay of all three pictures. (**c**) *In vitro* dye transfer in differentiated transgenic myotubes (EGFP^+^ , left); patch loading of the upper myotube (arrows) results in progressive dye transfer of Alexa 350 (middle left), but not of Alexa 546-dextran (middle right) into the neighbouring EGFP^+^ SkM (arrowheads). A brightfield image (right) shows a dense monolayer of differentiated and elongated myocytes. (**d**,**e**) Sirius Red staining of infarcted hearts 12–14 days after the lesion reveals engraftment of EGFP (**d**) and Cx43-EGFP (**e**) *ex vivo* transduced SkM (fibrotic tissue red, viable cells yellow) in the transmural scar area. Macroscopic imaging and quantitative morphometry revealed in average 19.185 ± 18.743 and 5.338 ± 4.552 engrafted cells in EGFP-SkM and Cx43-SkM engrafted hearts, respectively (n = 5 each). Insets show EGFP^+^ SkM (green, autofluorescence brown) or Cx43 immunostaining (red; nuclear Hoechst stain, blue), of engrafted Cx43-SkM (e, upper right). (**f**) Burst stimulation induces self-terminating VT in a representative EGFP-SkM transplanted mouse *in vivo*. (**g**) No VT is evoked in a representative Cx43-SkM transplanted mouse upon burst stimulation. (**f**,**g**) Top trace, surface ECG; bottom trace, atrial intracardiac lead; A, atrium; V, ventricle. (**h**) Statistics of VT incidence upon burst stimulation *in vivo* reveals prominent reduction of VT inducibility after engraftment of Cx43-SkM compared to EGFP-SkM (p < 0.01). Scale bars: b = 30 µm; c = 200 µm; d,e = 500 µm; d,e insets = 100 µm; e upper right inset = 10 µm.

To evaluate the ability of viral gene transfer to effect an increase in homotypic and heterotypic electrical conduction, we first explored the generation of gap junctions by lentivirus Cx43 transduction of skeletal muscle cells (SkM), as these cells do not express functional Cx43 gap junctions. Myoblasts were harvested from diaphragm and hindlimb muscles and transduced with lentivirus expressing either EGFP and the cardiac gap junction protein Cx43, or EGFP alone, under control of the cytomegalovirus (CMV) promoter (Fig. 1a). EGFP transduction efficiencies 48 hours after overnight virus exposure (MOI 25) were approximately 20% (489 cells analyzed) and 12% (1,080 cells) with EGFP and Cx43-IRES-EGFP constructs, respectively. Cx43 transduction efficiency is likely greater than indicated by EGFP expression due to incomplete translational efficiency of the IRES element; Cx43 immunostaining was prominent in the membrane of all EGFP^+^ cells (Fig. 1b). The formation of functional gap junctions in Cx43^+^ SkM was first assessed *in vitro* by localized electroporation of single SkM in a dense monolayer (Fig. 1c, right panel) with fluorescent dyes. As shown in Fig. 1c, dialysis of a Cx43/EGFP^+^ SkM cell (left panel) revealed diffusion of the Alexa 350 (349 Da; 1 ng/nl) dye into the neighboring EGFP^+^ SkM, whereas the larger Alexa 546 (10 kDa; 10 ng/nl) dextran-coupled-dye did not diffuse (middle panels; see also Suppl. Fig. 1b), excluding formation and diffusion through cytosolic bridges. Dye transfer into neighbouring Cx43/EGFP^+^ SkM cells could be observed in 28 of 75 successful electroporations. Importantly, dye transfer into adjacent Cx43/EGFP^−^ SkM could never be observed (0 of 75 successful electroporations), even though Cx43/EGFP^−^ SkM cells represented the large majority of cells in the monolayer. This internal negative control underscored that virus-based Cx43 expression and gap junction formation in skeletal myotubes was required for functional dye transfer. Next, we sought to determine whether lentivirus-mediated Cx43 transduction of SKM results in formation of functional gap junctions *in vivo* and hence post-MI VT protection equivalent to our earlier studies indicating that engraftment of SkM harvested from transgenic mice overexpressing Cx43 under control of a SkM promoter protected against post-MI VT *in vivo*. We have used the cryoinjury model, because it yields reproducible lesions, is transmural, and gives rise to re-entry^14^, which is the main mechanism responsible for VT induction also in humans. EGFP (EGFP-SkM) and EGFP-IRES-Cx43 (Cx43-SkM) transduced SkM (200,000 cells) were injected into cryoinjured mice immediately after the lesion and the electrical vulnerability tested *in vivo* 12 to 14 days later by applying burst and extrastimulus protocols (see also Suppl. Fig. 1a); then, hearts were harvested and analyzed. Engrafted EGFP^+^ cells were observed in 20 EGFP-SkM (80% of operated animals) and 15 Cx43-SkM (54% of operated animals) hearts 12-14 days after the operation (Fig. 1d,e); grafted cells were found to express typical SkM genes (Suppl. Fig. 1c). Lower (ca. 3.5 fold) engraftment rates were observed for Cx43-SkM, possibly due to viability issues because of hemichannels^16^. Representative ECGs from mice undergoing burst stimulation demonstrated that in the EGFP-SkM transplanted mouse a self-limiting VT (Fig. 1f) was evoked, whereas in the Cx43-SkM transplanted mouse no VT and a rapid return to normal sinus rhythm upon termination of stimulation could be observed (Fig. 1g, for prove of ventricular capture during burst stimulation see Suppl. Fig. 1d). Statistical analysis of the *in vivo* electrophysiological testing of mice with proven EGFP-SkM or Cx43-SkM grafts revealed that VT incidence was 70% (n = 20) in EGFP-SkM mice, whereas it was only 20% (n = 15) in Cx43-SkM (p < 0.01) engrafted mice (Fig. 1h). Besides the prominent quantitative reduction of VT incidence in C43-SkM engrafted mice, also VT severity was reduced, as most non self-limiting VT were never observed in this group (Suppl. Fig. 4). Thus, our data demonstrate functional gap junction formation and anti-VT protection upon lentivirus-Cx43 overexpression.

### Injection of lentivirus-Cx43 into the resolving myocardial infarct results in Cx43 overexpression in resident (myo)fibroblasts and CD45^+^ cells in the scar

**Figure 2:**
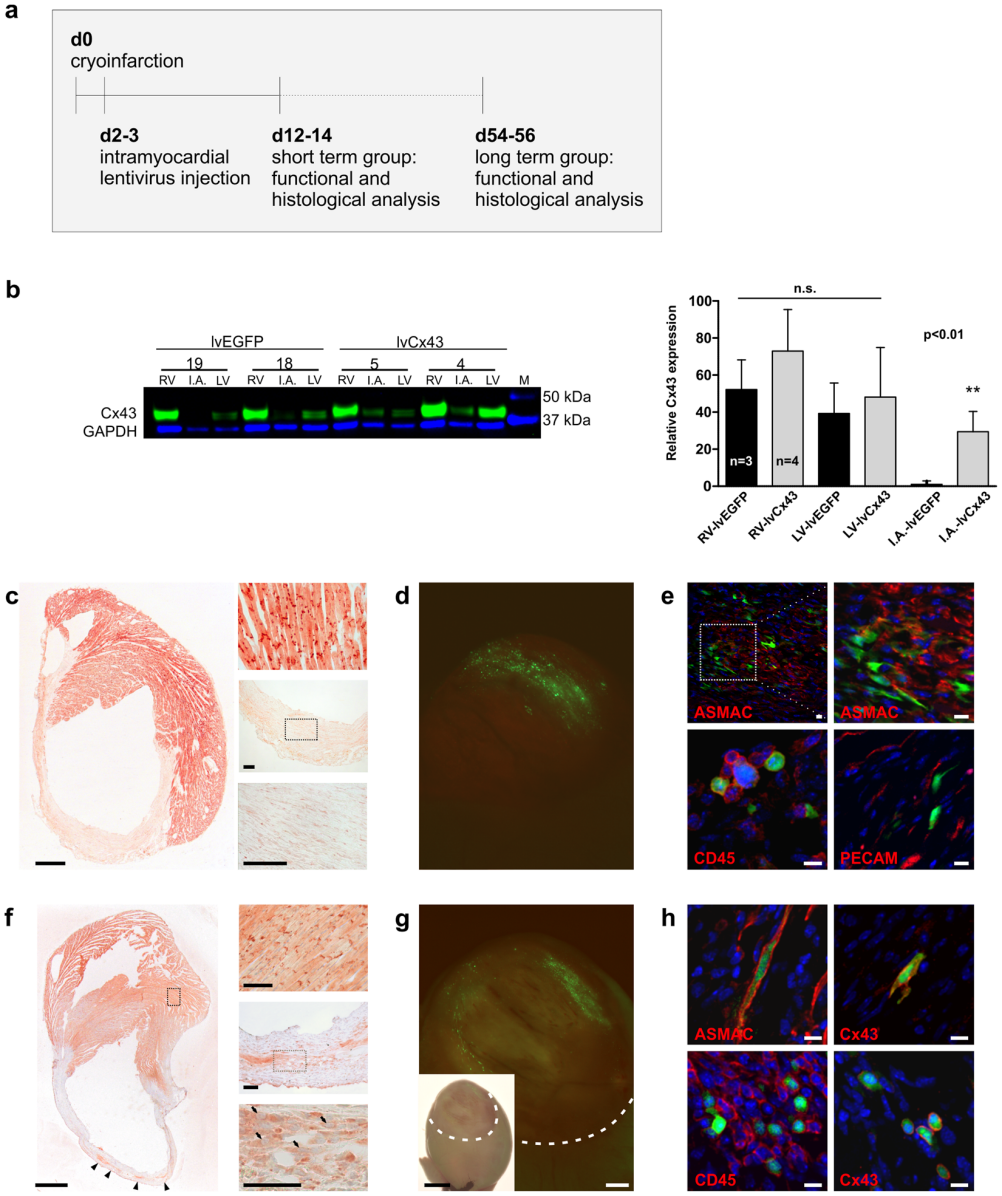
Cx43 and EGFP expression in the myocardial scar area after direct intramyocardial injection of lentivirus constructs 2–3 days after infarction. (**a**) Surgical protocol and time points of analysis after gene therapy. (**b**) Western blot analysis of infarct tissue harvested from hearts 12–14 days after virus injection proves expression of Cx43 after injection of lvCx43; note very weak Cx43 expression in lvEGFP hearts (original data are depicted in Suppl. Fig. 6). Quantitative analysis of Cx43 protein content yielded a 27,6 fold increase (p < 0.01) in lvCx43 vs lvEGFP injected mice 10–12 days after virus application. (**c**–**e**) lvEGFP injected heart. (**c**) Lack of Cx43 expression in the scar area after injection of lvEGFP into the infarct (AEC staining, red), whereas the native myocardium is strongly Cx43^+^; insets show magnification of remote (top) and infarct areas (middle, bottom). (**d**) Native EGFP fluorescence reveals transduction of cells within the scar region (infarct area labeled by dotted line). (**e**) Cell type-specific immunostainings (red; native EGFP, green; nuclear Hoechst stain, blue) against ASMAC (myofibroblasts, smooth muscle cells), CD45 (hematopoietic cells), and PECAM (endothelial cells) identify the transduced cell types; the upper right panel displays magnification of the boxed area in the upper left panel. (**f**–**h**) lvCx43 injected heart. (**f**) Injection of lvCx43 leads to scattered islets of Cx43 transduced cells (arrowheads) within the infarct area (AEC staining, red); insets show magnification of remote (top) and infarct area (middle, bottom); note nuclear stain (Hematoxylin, blue) of Cx43 expressing cells (arrows, bottom). (**g**) Native EGFP fluorescence and (**h**) immunostainings as described above; Cx43 stainings (red) reveal Cx43 protein expression in the membrane of ASMAC^+^ and CD45^+^, but not PECAM^+^/EGFP^+^ cells. Scale bars: c,f = 1 mm; c,f insets = 100 µm; d,g = 500 µm; d,g insets = 2 mm; e,h = 10 µm.

We next sought to determine the feasibility of direct Cx43-based gene therapy directed at resident cells of the myocardial scar area, as such a strategy would avoid the known technical and biological problems associated with cell therapy^17^. We employed lentiviral vectors, in contrast to AAVs, to ensure broad cellular tropism and permanent transgene expression, and targeted fibroblasts and other non-myocytes within an ablative infarct. Gene transfer was performed 2–3 days after the initial injury, a time point when fibroblasts are known to start to replace cardiomyocytes^18^ (Fig. 2a). To this end a single intracardiac injection (5 µl) of either lentivirus-EGFP (lvEGFP) or lentivirus-Cx43-IRES-EGFP (lvCx43) vector was administered into the lesion. We took advantage of cryoinjury as lesion type, because (i) lesion size is highly reproducible, (ii) no cardiomyocytes survive within the lesioned area^19^, and, most importantly, because (iii) intracardiac virus injection is, in contrast to left coronary artery ligation, feasible at 2–3 days after the initial lesion.

As shown in Suppl. Fig. 2a, 10–12 days after virus injection integrated lentiviral DNA could be detected using PCR within the lesion of all injected mice, but not in the right ventricle of the same animals (n = 3 for each construct), respectively. In addition, we also analyzed EGFP expression by qPCR and found that it was exclusively expressed in lvCx43- and lvEGFP injected scar areas, but not in native left- and right ventricles (n = 4 for each construct) of the same animals (Suppl. Fig. 2b). Next, we investigated Cx43 protein content in scar areas and in the native myocardium by Western Blot analyses. Quantitation revealed a 27,6 fold increase (p < 0.01) of Cx43 in infarct areas of lvCx43 (n = 4) vs lvEGFP-injected mice (n = 3) at 10–12 days after virus application (Fig. 2b). Relative Cx43 expression (normalized to GAPDH) within the scar area was, as expected, substantially lower than in the native right- or left-ventricular heart tissue (Fig. 2b); our Western Blot data do not provide information in regard to post-translational processing of Cx43. Western Blots revealed, as would be expected, prominent EGFP expression only in I.A. of lvCx43- and lvEGFP injected hearts (data not shown). EGFP fluorescence was directly observed within the infarct areas of both lvEGFP and lvCx43 injected hearts two weeks post-operation (Fig. 2d,g), however the EGFP signal was much weaker in the latter group, consistent with less efficient translation of the post IRES sequence cDNA. Cx43 immunostaining revealed a characteristic distribution pattern of scattered islets of Cx43 transduced cells (note nuclear stain in blue) in parts of the infarct area (Fig. 2f, lower panel). No expression of Cx43 was observed in the infarcted area of lvEGFP injected mice (Fig. 2c), despite typical expression in the surrounding myocardium.

To determine the cell type(s) expressing lentivirus genes, we stained hearts for different lineage specific markers. As expected, no cardiomyocytes were observed within the lesion following injury, and only one cardiomyocyte outside the lesion showed EGFP expression indicating that the lentiviral transduction was restricted to cells within the scar area. In fact, strong EGFP fluorescence was detected in other cell types within the infarct zone, these amounted to approximately 4000 cells upon lvEGFP and 1800 cells upon lvCx43 injection. When counting the cells in the scar based on Hoechst positive nuclei (n = 3 hearts), we estimated that approximately 1.1 to 2.8% of the resident cells were lentivirus-transduced.

The majority of EGFP^+^ cells co-localized with alpha smooth muscle actin (ASMAC) and displayed an elongated and branched fibroblast-like shape. In addition, numerous CD45^+^ mononuclear cells were EGFP^+^, whereas no EGFP^+^/PECAM^+^ endothelial cells were found (Fig. 2e). Similarly, although EGFP expression was reduced in lvCx43 hearts and fewer EGFP^+^ cells were detected, ASMAC^+^ and CD45^+^ (Fig. 2h), but not PECAM^+^ fluorescent cells, were observed (Suppl. Fig. 2c); Cx43 immunostaining showed clear membrane localization of Cx43 expression in EGFP^+^ cells (Fig. 2h). Thus, lentivirus-based gene transfer within days of cardiac injury constitutes despite the relatively low transduction rate an effective means of expressing cardiac gap junction protein Cx43 in endogenous cells within a resolving infarct.

### Gene therapy of the myocardial scar area with Cx43 causes a strong reduction of the post-MI VT incidence *in vivo*

**Figure 3:**
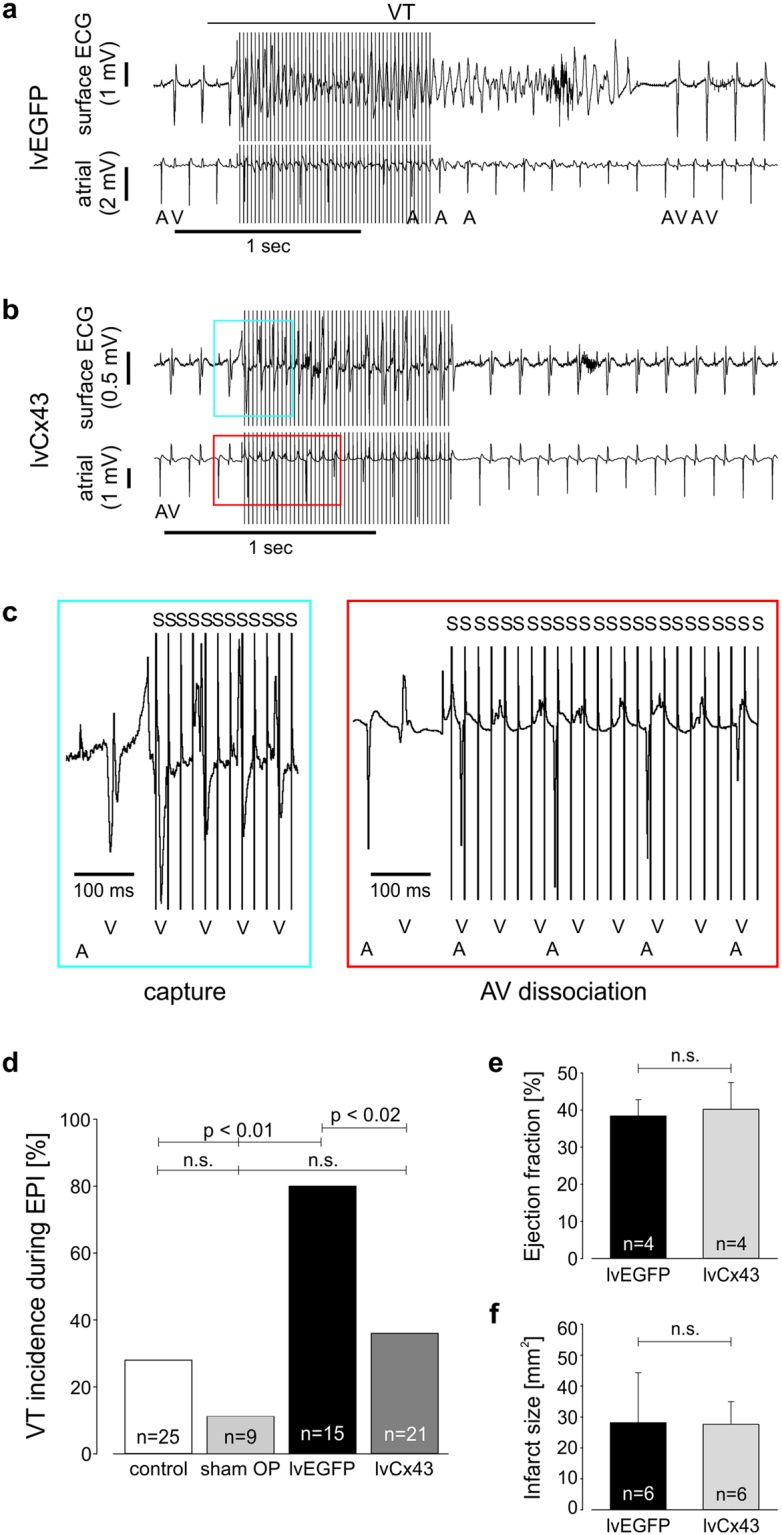
Injection of lvCx43 into the myocardial scar strongly lowers post-infarct VT incidence *in vivo* at 2 weeks after gene therapy*;* myocardial remodeling and left ventricular function remain unaltered. (**a**–**c**) *In vivo* electrophysiological testing. (**a**) Burst stimulation induces self-limiting VT with typical atrio-ventricular dissociation (lower panel) in a representative lvEGFP injected mouse (upper panel). (**b**) No VT is evoked in a representative lvCx43 transduced mouse upon burst stimulation. (a,b) Top trace, surface ECG; bottom trace, atrial intracardiac lead. (**c**) Magnification of traces shown in (b) reveals appropriate stimulation during the stimulation train, but no VT is induced; due to the short refractory period of murine ventricular myocardium a 3:1 capture during burst stimulation (S1S1 10–50 ms) is achieved. (a–c) A, atrium; V, ventricle. (**d**) *In vivo* VT incidence after transduction with lvCx43 is significantly reduced compared to lvEGFP injected control hearts (p < 0.02). (**e**,**f**) lvCx43 and lvEGFP injected hearts display very similar left ventricular function (**e**) and infarct size (**f**).

To determine whether Cx43 expression in non-muscle cells is sufficient to provide electrical protection, we examined VT vulnerability *in vivo* in lvEGFP and lvCx43 injected mice 10–12 days post gene transfer. Basic ECG parameters were not significantly different between the two lentivirally treated groups (Suppl. Table 1) or between IvCx43 injected mice and mice injected in a similar manner with lentivirally transduced SkM. In Fig. 3a, a representative ECG during burst stimulation of a lvEGFP treated animal shows the induction of VT in the early phase of the stimulation train; electrical instability is also seen in the surface ECG from this mouse (Fig. 3a upper trace), where persistently deformed and polymorphic QRS-complexes were observed, and in the intra-cardiac lead (Fig. 3a lower trace), in which the attendant atrio-ventricular (AV) dissociation (persisting rhythmic P-waves and irregular ventricular signals) is documented. The VT is spontaneously terminated and, after a compensatory pause, normofrequent sinus rhythm with narrow QRS-complexes, as prior to burst stimulation, is observed in the ECG traces. By contrast, despite effective burst stimulation and capture as indicated by the altered QRS-complexes and AV-dissociation during the stimulation train (Fig. 3c), rapid restoration to normal sinus rhythm without VT induction is observed in a representative lvCx43 mouse (Fig. 3b). Summary statistics of the *in vivo* electrophysiological testing revealed that 80% (n = 15) of lvEGFP-injected animals compared to 28% of uninfarcted control (n = 25) and 11% of sham operated (n = 9) CD1 wild type mice developed VT (p < 0.01, lvEGFP vs control or sham operated animals)(Fig. 3d). In contrast, significantly fewer lvCx43 than IvEGFP mice developed postinfarction VT (38%, n = 21; p < 0.02), and the incidence of VT initiation did not differ from CD1 wild type mice (p = 0.54). Moreover, more aggressive burst stimulation protocols were required to evoke VTs in those lvCx43 in which instability was observed (Suppl. Fig. 4). These data also demonstrate that the efficacy of VT protection after direct gene therapy is very similar to that provided by grafting Cx43 expressing muscle cells (reduction of VT incidence of 42% in lvCx43- and of 50% in Cx43-SkM injected mice compared to the respective controls). In contrast to SkM-EGFP engrafted mice, persistent VT were not observed upon direct intramyocardial lentiviral gene transfer further proving the pro-arrhythmic effect of myoblast grafts. In order to exclude that lentivirus-based Cx43 overexpression induces the anti-VT protective mechanism by indirect effects on infarct size and/or remodeling, we measured left ventricular function using *in vivo* catheterization and performed morphometric analysis of hearts. All functional parameters proved to be very similar between lvEGFP (n = 4) and lvCx43 (n = 4) injected hearts 10–12 days post gene transfer with an ejection fraction of 38.4 ± 4.4% *vs* 40.2 ± 7.2% (p = 0.70), a stroke volume of 23.3 ± 3.8 µl *vs* 20.7 ± 2.0 µl (p = 0.30) and a cardiac output of 12.2 ± 2.5 ml/min *vs* 9.8 ± 2.2 ml/min (p = 0.20), respectively (Fig. 3e, Suppl. Fig. 2d,e). Similarly, also infarct size 10–12 days after gene therapy was comparable between lvEGFP (n = 6) and lvCx43 (n = 6) injected mice with 28.8 ± 15.5 *vs* 28.5 ± 6.8 mm² (p = 0.97), respectively (Fig. 3f).

### Gene therapy of the myocardial scar area with Cx43 results in increased conduction velocities in the infarct and its border zone and long-time anti-VT protection

**Figure 4:**
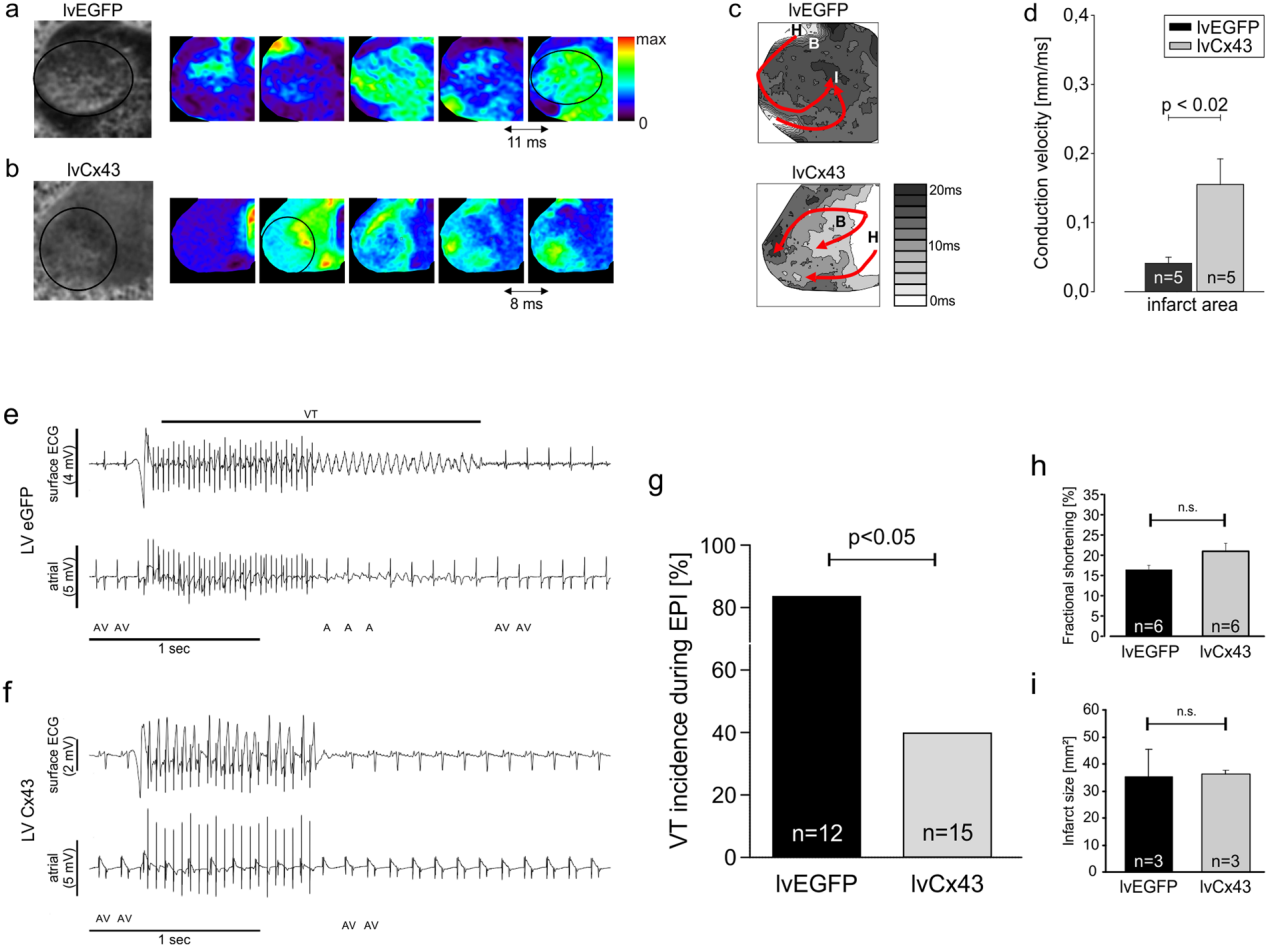
Injection of lvCx43 into the myocardial scar increases conduction in Langendorff-perfused hearts and provides long time protection against VT *in vivo*. (**a**–**d**) Optical mapping (**a**) Overview of a representative lvEGFP injected heart (leftmost panel, infarct region is encircled). Under sinus rhythm and unipolar pacing from the base of the heart, sequential di-4-ANNEPS fluorescence images display highly irregular and atypical conduction paths surrounding the lesioned area (images every 11 ms). (**b**) Also lvCx43 injected hearts revealed atypical propagation patterns, but some conduction through the scar area is visible (images every 8 ms). (**c**) Isochronal maps (same hearts as in a and b) depicting the activation wavefront and conduction delays near the infarct border zone. The activation bypasses the lvEGFP injected infarct (upper panel), but propagates through the lesioned area in the lvCx43 injected heart (lower panel); H, healthy; B, border zone; I, infarct. Scale bar indicates local activation times. (**d**) Local conduction velocities at 200 ms pacing. Note its significant increase in the infarct area (p = 0.0173) of lvCx43 injected hearts (n = 5) *vs* lvEGFP injected control hearts (n = 5). (**e**,**f**) Representative traces during *in vivo* burst stimulation 2 months after lentiviral gene transfer recorded from a lvEGFP (**e**) and a lvCx43 (**f**) injected heart. In the lvEGFP heart onset of VT shortly upon burst stimulation is observed (e), whereas no VT is induced in the lvCx43 heart (f); Top trace, surface ECG; bottom trace, atrial intracardiac lead; A, atrium; V, ventricle. (**g**) Statistical analysis of VT incidence shows a significantly (p < 0.05) lower incidence in the lvCx43 compared to lvEGFP control hearts. (**h**,**i**) There is no difference in left ventricular function (fractional shortening, **h**) and infarct size (**i**) between lvEGFP and lvCx43 injected hearts 8 weeks after gene therapy.

Our earlier work with engrafted embryonic cardiomyocytes^14^ indicated that the reduced VT vulnerability was associated with an increase in conduction velocity within the infarct zone. In order to explore the protective mechanism of our new Cx43 overexpression approach in the scar, we performed optical mapping in Langendorff perfused hearts 12–14 days after infarction. In line with earlier reports from our and other groups^14,20,21^, we could also observe clear voltage signals in the infarct zone (Suppl. Fig. 5a,b); their amplitude was, as reported earlier, approximately 30–40% of the signals from the non-infarcted myocardium (Suppl. Fig. 5c), but did not differ in between lvEGFP and lvCx43 hearts. We also detected highly irregular and complex propagation vectors around the infarct zone of lvEGFP mice compared to non-infarcted control hearts (n = 5, Fig. 4a,c upper panel). Furthermore, we observed that the cryoablated infarct zone produced large anatomical blocks promoting sustained re-entrant arrhythmias for dozens of beats before spontaneous termination (Supplementary Video 1). In clear contrast, lvCx43 transduced hearts (n = 5) displayed continuous waves of propagation across the scar area (Fig. 4b,c lower panel), thereby providing conduction pathways that short-circuited the larger anatomical block and re-entrant circuit (Supplementary Video 2). There was no evidence that focal activity initiated or sustained these arrhythmias given the large field-of-view and temporal resolution (2 ms) of the optical apparatus. To gain further insight into the mechanism underlying postinfarction VT protection we measured local conduction velocity within the infarction scar in which cardiac myocytes are not found (Fig. 2c,f). Conduction velocity was significantly increased (3.8 fold) in the scar areas of lvCx43 injected hearts (n = 5) *vs* lvEGFP injected control hearts (n = 5, Fig. 4d). These findings indicate that improved electrical coupling in non–myocytes within the scar underlies the enhanced VT protection and can be achieved by higher Cx43 expression in resident non-myocytes.

Finally, to further explore the potential therapeutic implications of conferring postinfarction electrical stability by gene transfer, we explored whether the VT protective effect of direct Cx43 transduction of the resolving infarct persists over time, particularly since profound morphological changes in the scar and the native myocardium occur in this time span^22,23^. Cx43 immunostaining of hearts harvested 8 weeks after lentiviral gene transfer showed islands of Cx43 transduced cells within the infarct area (Suppl. Fig. 3b), whereas no clear Cx43 signal was found in infarcts of lvEGFP transduced mice (Suppl. Fig. 3a), indicating that lentivirus gene transfer resulted in stable Cx43 expression. Electrical vulnerability assessed 2 months after lentivirus injection indicated that lvCx43 (40%, n = 15, p < 0.05) treated mice had a significantly lower VT-incidence compared to lvEGFP (83.3%, n = 12) injected mice (Fig. 4e–g, Suppl. Fig. 3c). Similar to the short-term experiments, in almost all lvCx43 injected vulnerable mice only the aggressive burst stimulation could induce short lasting VT, whereas many lvEGFP animals displayed VT upon programmed stimulation (Suppl. Fig. 4). To exclude indirect effects of Cx43 expression in the infarct, echocardiography was performed to assess left ventricular function. We found that fractional shortening (16.4 ± 1.1% *vs* 21.0 ± 2.0%, n = 6, p = 0.07, Fig. 4h) and left ventricular enddiastolic diameter (0.50 ± 0.03 cm *vs* 0.51 ± 0.03 cm, n = 6, p = 0.74, Suppl. Fig. 3d), key parameters of left ventricular function, were not significantly different between lvEGFP and lvCx43 treated mice. In addition, morphometric analysis indicated similar infarct sizes for both groups with 35.4 ± 9.9 mm² (lvEGFP, n = 3) *vs* 36.2 ± 1.6 mm² (lvCx43, n = 3), respectively (p = 0.89, Fig. 4i). The protective effect of Cx43 gene transfer was not associated with an anti-inflammatory effect on CD45^ +^ cell influx, as similar numbers of CD45^+^ cells were found in the infarcts of both groups (94.9 ± 39.6 cells/mm² (lvEGFP, n = 4) vs 50.7 ± 0.7 cells/mm² (lvCx43, n = 3)), and no difference was observed in the size of the lesions.

## Discussion

Herein we demonstrate that lentivirus-based overexpression of Cx43 in non-excitable cells within a resolving infarct markedly reduces post-infarct scar-related VT. This overexpression of Cx43 in the scar area leads to an increase of electrical conduction velocity between the native myocardium and the infarct area and this is most likely due, as reported earlier by other groups, to heterotypic cell-cell electrical coupling^24–27^.

The cardiac gap junction protein Cx43 plays a key role in the coordinated spread of electrical activity underlying sinus rhythm^28,29^. We and others have shown that engraftment of Cx43 expressing cardiomyocytes or genetically engineered skeletal myoblasts within an infarct is sufficient to dramatically reduce the incidence of post-infarct arrhythmias *in vivo*, suggesting that the absence of Cx43 within the scar underlies much of the observed risk of sudden death following ischemic cardiac damage^14,30^. However, there remain unique challenges to cell-based therapies in the working heart^16,31,32^, as engraftment rates rapidly decline following transplantation^17^, and may be pro-arrhythmic^4,14,33^. Similarly, direct re-programming approaches of resident fibroblasts in the scar remain inefficient^34^ and the underlying mechanisms and potential complications are poorly understood.

We therefore focused on the feasibility of direct gene therapy-based overexpression of Cx43 within resident cells or cells that migrate to the area of injury immediately post infarction, as such a simple strategy could be potentially translated into the clinic for the treatment of post-infarction scar-related VT. A recent study in pigs demonstrating transient expression of Cx43 in border zone cardiomyocytes following transcoronary perfusion of AAV further supports this approach^35^. We reasoned that direct virus application enhancing heterotypic cell-cell coupling within non-excitable cells of the infarct region, where even modest increases in conduction velocity have been reported to have a major effect on electrical vulnerability^14^, would constitute a simple and effective approach to reduce post-infarction arrhythmia vulnerability. We focused on the time period during scar formation and up to two months thereafter, as in post-infarct patients electrical vulnerability is high and clinical intervention often initiated. In addition, we chose to target only the infarct region in an effort to optimally match electrical properties across the infarction. This approach concentrates and restricts expression to the area devoid of cardiac connexin expression, whereas expression of the transgene predominantly in cardiomyocytes and/or border zone cells might not be sufficient to avoid central electrical mismatches. Furthermore, as border zone areas become less relevant because of re-perfusion therapy, recent clinical electrophysiology strategies also target directly the scar area instead of the border zone. Using this approach, robust Cx43 expression was observed in resident myofibroblasts and CD45^+^ cells populating the center of the scar area, cell types that till now could not be efficiently transduced with adeno-virus or AAVs. Even though the expression pattern was inhomogeneous, VT-incidence was consistently reduced, confirming our earlier findings that engraftment of low numbers of Cx43 expressing cells suffices to strongly reduce electrical vulnerability^14^. In future studies multiple virus injections into the scar area and/or the combination of nanoparticles with magnets could be probed to further increase transduction efficiency of resident cells and potentially also the anti-VT effect^36^. Direct lentivirus delivery did not affect cardiac function and remodeling, as basic ECG- and morphometric parameters were unchanged following injection; accordingly, we could not find lentivirus transduction of non-infarcted heart tissue. Our voltage mapping data clearly showed slow (0.05 mm/ms) electrotonic conduction of electrical activity into the scar region, even though in-depth histological analysis excluded, in contrast to other cardiac lesion types and animal models, surviving CMs within the lesion^14,19^. We speculate that the electrical activity within the scar is generated most likely from myofibroblasts, which are able to conduct electrical activity over distances as long as 300 µm *in vitro*^24,25,37^. In fact, a recent report on electrotonic conduction across myocardial scar tissue in mouse underscores the functional relevance of gap junctions, because knock-out of Cx43 in fibroblasts strongly reduced voltage mapping signals in the lesion^20^. In addition, elegant studies using voltage sensitive fluorescent protein expression in cardiac non-myocytes underscore heterotypic cell-cell coupling^26^.

Thus, our findings suggest that Cx43-overexpression in myofibroblasts destabilizes re-entry circuits around the infarct by augmented electrotonic conduction of electrical activity through the infarct scar^38–40^. A remarkable finding is the marked functional impact of modest increases in length constant and conduction velocity. While the small size of the mouse heart may exaggerate this functional effect, it may be reasonable to assume that the reduction of conduction inhomogeneity within local areas of larger hearts may have an important effect on arrhythmia, this needs to be tested in large animal models. We cannot exclude that Cx43 overexpression could also have additional effects besides lowering intercellular resistance or membrane potential, as gap junctional coupling enhances the exchange of signaling molecules. This and/or also Cx43 lateralization could alter the myofibroblast phenotype or paracrine function, which have been reported to affect cardiac arrhythmias^41,42^. In addition, also border zone cardiomyocytes and their coupling via Cx43 and/or Na_v_ channels could be affected by our manipulations of the scar area. Our data in lvCx43 hearts suggest that permanently transduced (myo)fibroblasts remain part of the scarred myocardium. This is underscored by the *in vivo* electrophysiology data at 2 months after gene therapy demonstrating that the protective anti-VT effect is relatively long lasting. Besides myofibroblasts, we found that also CD45^+^ immune cells within the scar area are Cx43 transduced. Given that neither lesion size, nor left ventricular function, nor the number of CD45^+^ cells strongly differ between controls and Cx43 transduced hearts, it is unlikely that these cells play an important role in VT protection. In summary, Cx43 gene therapy by transduction of resident cells within the damaged myocardium, results in effective suppression of VT risk through the induction of heterotypic cell-cell coupling. We demonstrate that a relatively simple strategy consisting of a single injection of lentivirus into the damaged myocardium constitutes an effective and long lasting way to manage the substantial risk of fatality associated with acute myocardial infarction. These data also highlight the concept of non-myocyte electrical conduction as a key target in post-infarction cardioprotection.

## Methods

### Experimental design

Aim of this study was to explore Cx43 gene therapy in the myocardial scar as means to reduce post-infarct VT incidence. For this purpose we tested first the lentivirus (lentivirus-EGFP or lentivirus-Cx43-IRES-EGFP) constructs in regard to the expression of functional Cx43 gap junctions *in vitro* and also *in vivo* using grafting of *ex vivo* transduced SkM. Next, we established a surgical protocol for direct lentiviral transduction of resident cells in the infarct area 2–3 days after the lesion. Electrical vulnerability was assessed at 2 or 8 weeks after the primary surgery by *in vivo* electrophysiological testing. For the analysis of conduction velocity *ex vivo* voltage mapping (2 weeks postoperatively) was performed. Cardiac function was evaluated by left ventricular catheterization or echocardiography, following functional experiments the hearts were harvested and further processed (see also Fig. 2a and Suppl. Fig. 1a).

### Virus generation

For production of self-inactivating (SIN) lentiviral vectors (LVs), HEK293T (human embryonal kidney cells) helper cells were co-transfected with the lentiviral plasmid and the packaging plasmids pMDLg/pRRE, RSV-rev^43^, and pMD2.G^44^. Recombinant replication deficient LVs of the 3^rd^ generation were purified from cell culture supernatant using ultracentrifugation as described elsewhere^45^. Briefly, producer cells were seeded on poly-L-Lysine coated cell culture dishes in DMEM (Invitrogen, Darmstadt, Germany), supplemented with 10% fetal calf serum (Biochrom, Berlin, Germany), 100 U/ml penicillin/100 μg/ml streptomycin (Pen/Strep; Biochrom, Berlin, Germany) and incubated at 10% CO_2_ and 37 °C. Cells were transfected at ~50% confluency by calcium phosphate transfection and subsequently incubated at 3% CO_2_ and 37 °C overnight. The medium was changed the next day and cells were cultured again at 10% CO_2_ and 37 °C. For purification of the vesicular stomatitis virus glycoprotein G pseudotyped LVs, cell culture supernatant was collected one day after medium change. Cells were incubated again with fresh medium and virus containing supernatant was collected again on the next day. The supernatants were filtered using a bottle-top filter (SFCA, 0.45 μm, Nalgene, Thermo Fisher Scientific, Waltham, MA, USA) to remove cell debris, transferred to centrifugation tubes and centrifuged in an ultracentrifuge with SW32Ti rotor (Optima L-100 XP, Beckman Coulter Incorporated, Brea, CA, USA) for two hours at 19,400 rpm and 17 °C, respectively. The virus pellets were re-suspended in HBSS (Invitrogen, Darmstadt, Germany) and virus from the first harvest was stored overnight at 4 °C to be combined with the virus from the second harvest. The combined pre-concentrated virus suspension was layered on top of a 20% (w/v) sucrose cushion and ultracentrifuged in a SW55 rotor (Optima L-100 XP, Beckman Coulter Incorporated, Brea, CA, USA) for two hours at 21,000 rpm and 17 °C. The LV pellet was resuspended in HBSS and vortexed for 45 min at 1,400 rpm and 16 °C. After a short spin down of debris for 3 s at 16,000 g the opaque supernatant was aliquoted and LV aliquots were stored at −80 °C.

The original lentiviral plasmids were derived from the lab of Inder Verma (The Salk Institute for Biological Studies, Laboratory of Genetics, La Jolla, CA, USA). The control vector rrl-CMV-EGFP contained a CMV promoter driven EGFP expression cassette. For production of Connexin 43 (Cx43) expressing LVs, the construct rrl-CMV-Cx43-IRES-EGFP was cloned containing a CMV promoter driven murine Cx43 expression cassette combined with an internal ribosome entry site (IRES) for simultaneous EGFP expression. Both constructs contained a central polypurine tract to increase the nuclear transport of the virus pre-integration complex and thus the transduction efficiency^46^ and the post-transcriptional regulatory element of woodchuck hepatitis virus (WPRE) to increase transgene expression^47^.

### Titration of lentiviral vectors

In order to determine the biological titer of LVs (in infectious particles (IPs) per ml) HEK293T cells were seeded in a 24 well plate. After cell attachment, they were transduced with the vector preparation of serial dilutions in 300 µl supplemented medium and incubated overnight at 10% CO_2_ and 37 °C as already described^45^. Medium was added on the next day. 72 h after transduction, cells were trypsinized and subsequently fixed with 4% (w/v) paraformaldehyde for 15 min on ice. Cells were centrifuged (5 min, 300 g) and re-suspended in phosphate buffered saline. Fixed cells were analyzed using flow cytometry and percentages of EGFP positive cells were determined. Non-transduced cells served as control. The biological titer was determined according to the following formulas: MOI (multiplicity of infection) = −ln (percentage of EGFP-negative cells/100); IP/ml = (number of infected cells) × (MOI) × (dilution factor) × 1,000. The biological titers of LVs were in the range of 1 to 10E + 09 IP/ml and were determined individually for each virus batch. The physical titer of LV preparations was determined by use of a commercially available colorimetric reverse transcriptase assay (Roche Diagnostics, Indianapolis, IN, USA) quantifying active viral reverse transcriptase (RT). The physical titers of LV preparations were in the range of 5 to 20 µl per 200 ng RT and were measured for each virus batch as well.

### Lentiviral *in vitro* transduction of cells

For *in vitro* tests, 50,000 HEK293T cells were plated in a 24 well dish. 24 h after seeding, cells were transduced with LVs in 300 µl medium overnight. On the next day, medium was filled up. 48 h post infection, cells were analyzed using fluorescence microscopy. The volume of LV preparation was calculated according to the biological titer and the MOI desired according to the following formula: LV volume [µl] = number of cells seeded × MOI/biological titer [IP/µl]. In compliance to the used volume and the physical titer of the virus batch ng RT applied could be determined.

### Generation of transgenic myoblasts by lentiviral gene transfer

Skeletal myoblasts (SkM) were harvested from the diaphragm and the hindlimb muscles of E18.5–19.5 CD1 wildtype embryos. Muscles were isolated by blunt dissection and enzymatically digested with collagenase B (1 mg/ml, Roche Diagnostics, Indianapolis, IN, USA) and trypsin (0.05% (w/v), Gibco/Life Technologies, Darmstadt, Germany) at 37 °C for 60 minutes. Then, the cell suspension was filtered through a 100 µm cell strainer and isolated cells were plated for several hours on fibronectin coated 24 well plates (250,000 cells/well) in 300 µl IMDM containing 20% FCS (Gibco/Life Technologies, Darmstadt, Germany) at 37 °C. For lentiviral transduction the virus constructs (CMV-EGFP or CMV-Cx43-IRES-EGFP) were added at a MOI of 25 overnight at 37 °C. The day after cells were washed at least twice with PBS and 1 ml IMDM/FCS was added.

Prior to intramyocardial injection the cells were plated for additional 24 hours, then trypsinized and re-suspended in IMDM/FCS at a concentration of 40,000 cells/µl. SkM, which were not injected, were re-plated and their differentiation *in vitro* monitored for several days. For *in vitro* coupling experiments, cells were cultivated for up to 20 days in IMDM/FCS. To avoid fibroblast overgrowth cells were irradiated with 15 Gy at the 3^rd^ day of cell culture^48^.

### Dye dialysis to prove functional gap junctions after lentiviral Cx43 gene transfer

To analyze functional coupling between transduced SkM, single-cell-electroporation was used. Sharp electrodes, filled with 40 mM KCl and two different dyes (Alexa350 and Alexa546-Dextran) were attached to the cell membrane of cultured (10–20 days) myoblasts resulting in a resistance of 140–190 MΩ. Alexa350 (349 Da; 1 ng/nl, Life Technologies, Darmstadt, Germany) emits blue fluorescence and is a small molecule, which is able to pass through Cx43 gap-junctions between adjacent cells. Dextran coupled Alexa546 (10 kDa; 10 ng/nl, Life Technologies, Darmstadt, Germany) emits in the red region of the spectrum and diffuses only to neighbouring cells through cytoplasmic bridges because of its high molecular weight^49^. Next, the cell membrane was perforated by applying an alternating current of 400 Hz and 10–50 nA in square-pulses and the electroporated myotubes were allowed to load via iontophoresis with the two different dyes. Dye transfer to adjacent EGFP^+^ cells was observed using fluorescence microscopy (Axiovert 200, Carl Zeiss, Jena, Germany) in 28 EGFP^+^ cells, dye transfer into an EGFP^-^SkM was never observed.

### Intramyocardial transplantation of Cx43 expressing myoblasts

Cryolesions were generated in 53 adult female CD1 WT mice (age 10–12 weeks). The free left ventricular wall of the heart was exposed by a left lateral thoracotomy under inhalative anesthesia (endotracheal intubation and ventilation, 40% O_2_, 60% N_2_O, 1.5–2.0 Vol% Isoflurane) and a liquid nitrogen precooled copper probe (diameter 3.5 mm) was attached to the surface of the heart (three times, 10–15 seconds). Then, 200.000 myoblasts transduced with CMV-EGFP (n = 25) or CMV-Cx43-IRES-EGFP lentivirus (n = 28) were re-suspended in 5 µl culture medium and injected between the center and the border zone of the acute cardiac lesion using a 10 µl Hamilton syringe equipped with a 29 G insulin needle. To the cell suspension a food dye was added to visualize diffusion of the injected cell suspension from the center to the border zone of the lesion, otherwise a second injection nearby was applied. Afterwards, the chest was closed in layers, the pneumothorax evacuated by a chest drain and the animals allowed to awake, as reported before^23^.

### Direct lentiviral Cx43 transduction of the cardiac lesion *in vivo*

For direct lentiviral gene transfer of cells in the scar area, 81 CD1 wildtype mice (see also above) were cryoinjured, then 2–3 days after the first operation, the first thoracotomy was surgically re-opened under general anesthesia and the myocardial lesion visualized. Then, *via* a 10 µl Hamilton syringe equipped with a 33 G insulin needle 5 µl of lentivirus solution (CMV-Cx43-IRES-EGFP, n = 36 or CMV-EGFP, n = 45) was applied by a single injection between the center and the apical margin of the lesion. The infectious particles amounted to 5.8 × 10^8^ to 3.6 × 10^9^ per ml for the CMV-Cx43-IRES-EGFP lentivirus and 1 × 10^9^ to 1.5 × 10^10^ per ml for the CMV-EGFP lentivirus. Thereafter, the chest was re-closed and the mice allowed to wake up. Peri- and postoperative analgesia (0.1 mg/kg Buprenorphin 2 ×/d and 5 mg/kg Carprofen 1 ×/d s.c.) was administered to all operated animals up to the third postoperative day after each surgery. Sham operations were performed in 9 mice: First, a lateral thoracotomy was performed, the pericardium opened and the chest re-closed. Then, in analogy to virus-injected mice a second re-thoracotomy was performed two days after the first surgical intervention. Mice of the *ex vivo* imaging and long term groups were treated additionally with Dexamethasone 0.02 mg 2 ×/d s.c up to 7 days.

### *In vivo* electrophysiology, left ventricular catheterization and echocardiography

*In vivo* electrophysiological testing was performed under inhalative anesthesia (Isoflurane 1.0 to 1.5 Vol %) 12–14 and 56 days after generation of the myocardial lesion by a blinded investigator. As reported before^14^, a surface 6-lead ECG was recorded (PowerLab 16/30, LabChart 7, ADInstruments, Pty LTD, Australia), then the tip of a 2 French octapolar mouse-electrophysiological catheter (CIBER Mouse Electrophysiology Catheter, NuMED, USA) was inserted to the apex of the right ventricle via the right jugular vein. Bipolar intracardiac electrograms were recorded from neighboring electrode pairs at the atrial, his-bundle and ventricular level. Rectangular stimulus pulses at twice pacing threshold were administered by a multi programmable stimulator (Model 2100, A-M Systems, USA; Stimulus 3.4 Software, Institute for Physiology I, University of Bonn, Germany) *via* the apical electrode pair. To explore electrical vulnerability both, extrastimulus protocols and the more aggressive burst stimulus were used. The pacing threshold current (1 ms stimulus duration) was between 0.5 and 1.0 mA and rectangular stimulus pulses of 2-fold pacing threshold were applied. Ventricular vulnerability was tested as reported earlier^14^ by extrastimulus pacing with up to three extra beats (with 10 ms stepwise S1S2 or S2S3 reduction starting 10 ms beneath S1S1) at S1S1 cycle lengths of 120 ms, 100 ms, and 80 ms. Next, ventricular vulnerability was also tested by applying ventricular burst stimulation: S1S1 stimulation at cycle lengths starting at 50 ms with 10 ms stepwise reduction down to 10 ms were performed for 1 second each and repeated 3 times. Between stimulation procedures the hearts were allowed to recover for at least 10 seconds. For the analysis of the *in vivo* electrophysiology data we have used, as reported in earlier publications^50^ and also by our groups in Bonn^51,52^, the clinical definition of VT, namely 4 consecutive ventricular extrabeats with atrioventricular dissociation. Given the high heart rate and the short refractory period in mice, more aggressive stimulation protocols than in humans need to be applied. Electrical noise due to motion and/or other artifacts was reduced by appropriate filtering (10–100 Hz) of the data. Upon VT induction both, mono- and polymorphic VT could be observed.

For *in vivo* left ventricular catheterization inhalative anesthesia was used as described above. A 1.4 French Millar Aria1 catheter (Millar Instruments Inc., Houston) was inserted retrogradely into the left ventricle via the right carotid artery and pressure-volume loops were recorded with PowerLab 16/30, LabChart 7 (ADInstruments, Pty LTD, Australia) and analyzed by the integrated PVAN-Software.

In long term experiments left ventricular function was measured 1–2 days prior to *in vivo* electrophysiological testing under inhalative anesthesia with M-mode echocardiography using a HDI-5000 ultrasound system (ATL–Phillips, Oceanside, CA, USA) equipped with a linear array 15 MHz transducer (CL15–7)^53^. In the parasternal short-axis view, M-mode data were acquired at the level of the papillary muscle and morphological as well as functional parameters measured as described before^54^.

### Histology, immunohistochemistry, and morphometry

After electrophysiological testing mice were heparinized and sacrificed, hearts harvested and imaged with a fluorescence zoom microscope (Axio Zoom V16, Zeiss). Then, hearts were mounted on a Langendorff perfusion apparatus, fixed by perfusion with 4% paraformaldehyde, cryopreserved in 20% sucrose and cut into 10 µm thick slices.

For evaluation of fibrosis and cell engraftment, Sirius Red staining was performed following standard protocols or engrafted cells were identified by their native EGFP fluorescence. Expression of Cx43 in virus-injected hearts was detected by immunohistochemistry after antigen retrieval using a customized polyclonal rabbit Cx43 antibody (PSL GmbH, Heidelberg, Germany). Staining was accomplished using the Vectastain Elite ABC system with AEC substrate (Vector Laboratories, Burlingame, CA, USA) and hematoxylin counterstain. To specify the cell type(s) expressing lentivirus genes we performed fluorescent immunostainings in one heart per virus-construct (20 sections each) for different lineage specific markers. Antibodies against sarcomeric alpha-actinin, alpha-smooth muscle-actin (ASMAC; Sigma-Aldrich, St. Louis, Missouri, USA), Connexin 43 (Cx43; PSL GmbH), CD45 (Lab Vision, Fremont, CA), MyoD (Dako, Hamburg, Germany), and platelet/endothelial cell adhesion molecule (PECAM; BD Pharmingen) were used as previously described^23^. Visualization was accomplished with appropriate secondary donkey antibodies conjugated to Cy3 or Cy5 (Jackson ImmunoResearch, West Grove, PA). Nuclei were stained with Hoechst dye 33342 (Sigma-Aldrich). Native EGFP fluorescence and immunofluorescence was imaged with a Zeiss Axiovert 200 microscope equipped with an ApoTome and an AxioCam MRm. Images were acquired by use of the AxioVision software (Zeiss).

For morphometric analysis hearts were perfused with cardioplegic solution (HTK Solution, Dr. Franz Köhler Chemie GmbH, Bensheim, Germany), cryopreserved and sectioned at intervals of 300 µm, as described above. Infarct size was determined in clearly relaxed hearts with transmural lesions by measuring the circumference of the damaged area based on autofluorescence; total infarct area was calculated by extrapolation of these measurements.

To quantify cellularity within the infarct area three sections at different levels (lower, mid and upper part of the lesion) were taken from two hearts at 14 days after infarction and stained with Hoechst dye. Tile images were recorded by AxioVision MosaiX and measurements performed using AutMess software (Zeiss). Infarct area was determined based on autofluorescence, and nuclei in this area were counted. Cellularity is given as nuclei per mm^2^. The number of EGFP^+^ cells was determined in ten hearts after SkM transplantation (five per construct) and in two hearts (one per construct) after pure lentivirus injection; 6 slides (3 sections each) spanning the complete engrafted area were analyzed by counting of nucleated (Hoechst dye stain) EGFP^+^ cells and extrapolating numbers to whole hearts.

### Analysis of Cx43 expression by western blotting after lvEGFP or lvCx43 injection into the infarct area

Infarct areas (I.A.), into which lvEGFP or lvCx43 was injected, and remote tissue of respective right and left ventricles were excised 10–12 days after lentiviral transduction under a fluorescence stereomicroscope (Leica MZ 16 F, Leica Microsystems) and frozen in liquid nitrogen. Heart tissues were homogenized in RIPA buffer (2 mM EDTA; 25 mM Tris-HCL, pH7.5; 150 mM NaCl; 0.1% Sodiumdoxycholate; 0.1% SDS; 1% Nonidet P40 in H_2_O; 2.5 × cOmplete Proteaseinhibitor, Roche). SDS mini gels (12% separating gel: 12% Acrylamide, 7.5 mM Tris-HCL (pH8.8), 0.1% SDS, 0.05% APS, 0.05% TEMED; 8% stacking gel: 8% Acrylamide, 1.25 mM Tris-HCL, 0.1% SDS, 0.05% APS, 0.1% TEMED) were prepared and protein lysates separated by electrophoresis (ProSieve EX Running Buffer 10x , diluted in H_2_O to 1x , Lonza; Precision Plus Protein WesternC Standards, Biorad), and blotted onto methanol-activated PVDF membrane (Low-fluorescence, 0.2 µm, Biozym) by tankblot (ProSieve EX Western Blot Transfer Buffer 10x , diluted in H_2_O to 1x , Lonza). Membranes were blocked with TBST buffer (20 mM Tris, 150 mM NaCl, 0.1% Tween-20 in H_2_O) containing 5% milk powder (Skim milk powder, VWR) for 1 hour. Cx43 (1:3000, custom-produced rabbit polyclonal antibody, PSL GmbH, Heidelberg, Germany) and horseradish peroxidase (HRP) conjugated-GAPDH (1:5000, Sigma) antibodies were incubated over night at 4 °C, followed by incubation of donkey-anti-rabbit Alexa Fluor 488 conjugated antibody (1:3000, AffiniPure, Jackson Immuno Research) and Precision Protein StrepTactin-HRP Conjugate (1:3000, Biorad) for 1 h at RT. Signals were developed using Pierce ECL Western Blotting Substrate (Thermo Scientific) and detected with ChemiDoc MP Imaging System (Biorad). All antibodies were diluted in 5% milk powder in TBST. Quantification of Cx43 expression was performed using Image Lab Software (Biorad) normalized to GAPDH.

### Determination of provirus integration in mouse hearts using a PCR assay after I.A. injection of lvEGFP or lvCx43

Infarct areas with virus injection (I.A.) of either lvEGFP or lvCX43 and areas from the respective right ventricle (RV) were excised 10–12 days after lentiviral transduction. Using the Puregene Core Kit A from QIAGEN (Mat. No. 1042601), genomic DNA was isolated according to the supplier. The forward (fwd) and reverse (rev) primer sequences (Eurofins MWG, Ebersberg, Germany) used for detecting integrated provirus DNA were as follows: 5′-TGTGTGCCCGTCTGTTGTGT-3′ (fwd) and 5′-GAGTCCTGCGTCGAGAGAGC-3′ (rev). PCR reactions were performed on a T-Professional Trio Thermocycler (Biometra). The 139 bp amplification product was stained with ethidium bromide.

### Analysis of EGFP expression in mouse hearts by qRT-PCR after I.A. injection of lvEGFP or lvCx43

I.A. of mouse hearts injected with lvCx43 or lvEGFP as well as respective remote areas were excised and stabilized in RNAlater (Qiagen). RNA was extracted following the standard Trizol protocol (Life Technologies) and RNA quality was determined using the Bioanalyzer 2100 (Agilent). RNA was reverse-transcribed and pre-amplified using the Cell Direct kit (Invitrogen) followed by qPCR (CFX96 cycler, Biorad). As controls, H_2_O and non-RT samples were used. TaqMan probes: GAPDH (4352932E), EGFP (custom-designed), TaqMan Gene Expression Master Mix (all from Applied Biosystems).

### *Ex vivo* optical mapping

Hearts were perfused in a Langendorff apparatus, mounted in a custom-designed chamber with a window for optical mapping, side restraints to minimize motion and temperature control to stabilize action potential durations and heart rate. Hearts were loaded with the voltage-sensitive dye di-4-ANEPPS (λ_ex_ = 540 ± 30 nm, λ_em_ > 640 nm) as previously described^55^. Voltage signals were recorded with a complementary-metal-oxide semiconductor (CMOS) camera (100 pixels × 100 pixels, Ultima-One SciMedia) at 1,000 frames per second. Conduction velocities within the infarct, border- and remote zones of myocardium were evaluated during pacing with a unipolar electrode. Light from a custom-designed 300 W tungsten-halogen lamp (University of Pittsburgh machine and electronic shops) was collimated, passed through an interference filter (540 ± 30 nm) and refocused to illuminate the heart. Fluorescent light from the heart was collected, passed through a high-pass filter (>640 nm) and was refocused on the CMOS camera, which viewed the anterolateral surface of the heart. In this optical alignment, optical maps included the complete lesion, border zone and adjacent native or ‘normal’ myocardium. With this optical configuration, the spatial resolution was close to cellular resolution (70 × 70 µm^2^ with a depth field of about 130–140 µm). To reduce motion blurring caused by rapid wave propagation the integration time was set to 1 or 2 ms. For stimulation of the hearts, an Ag^+^/AgCl electrode (200 µm in diameter) was pinned to the native myocardium, proximal to the lesion. Stimulation (impulse duration 2 ms, current 20% above threshold) was performed at a cycle length of 200 ms for at least 20 beats. Activation time-points were determined by dF/dtmax after applying a Savitzky–Golay smoothing filter (11 point width, third order), and isochronal maps of activation were generated as described previously. The local conduction velocity vector was calculated for each pixel by measuring the activation time-point at that pixel and comparing it with the activation time-points of its eight nearest neighbours. The rise time of action potentials was measured from fluorescence signals that showed a signal-to-noise ratio of more than about 20:1, at a sampling rate of 1,000 frames s^−1^ and a spatial resolution of 100 × 100 µm^2^ per pixel. The rise time was measured from 10% to 90% of action-potential amplitude from at least five consecutive action potentials and low-pass filtered with an averaging kernel of 1 ms. Border zones were identified visually from autofluorescence images.

### Statistics

Electrophysiological data were compared using a multivariant one-way analysis of variance with post hoc subgroup testing, where appropriate (Tukey-Kramer multiple comparisons test). For discrete variables a two-sided Fisher’s exact test was performed. Statistical evaluation of the other data was performed using Student’s t-test. A p value ≤ 0.05 was considered statistically significant, error bars represent SDs or SEMs (functional data).

### Study approval

All animal experiments were performed in accordance with National Institutes of Health animal protection guidelines and were approved by the local authorities (Landesamt für Natur, Umwelt und Verbraucherschutz, Nordrhein-Westfalen).

### Data availability

All datasets generated during the current study are available from the corresponding author on reasonable request and the results from all data analyzed during this study are included in the published article.

## Electronic supplementary material

Supplementary Materials

Supplementary Video 1. Voltage mapping after lvEGFP transduction

Supplementary Video 2. Voltage mapping after lvCx43 transduction
