# Supplementary material for: Overexpression of Cx43 in cells of the myocardial scar: Correction of post-infarct arrhythmias through heterotypic cell-cell coupling
Source: Sci Rep. 2018 May 8;8:7145. doi: 10.1038/s41598-018-25147-8 (PMC5940892; doi:10.1038/s41598-018-25147-8)

Supplementary Materials

Overexpression of Cx43 in cells of the myocardial scar: Correction of post-infarct arrhythmias through heterotypic cell-cell coupling

Wilhelm Roell[1*], Alexandra M. Klein[1,2], Martin Breitbach[2], Torsten S. Becker[2], Ashish Parikh[4], Jane Lee[3], Katrin Zimmermann[5], Shaun Reining[3], Beth Gabris[4], Annika Ottersbach[1,2], Robert Doran[3], Britta Engelbrecht[1,2], Miriam Schiffer[1,2], Kenichi Kimura[1,2], Patricia Freitag[2], Esther Carls[1,2], Caroline Geisen[2], Daniel G. Dürr[1], Philipp Sasse[2], Armin Welz[1], Alexander Pfeifer[5], Guy Salama[4], Michael Kotlikoff[3], Bernd K. Fleischmann[2*]

[1] Department of Cardiac Surgery, University of Bonn, Sigmund Freud Str. 25, 53127 Bonn, Germany

[2] Institute of Physiology I, Life&Brain Center, Medical Faculty, University of Bonn, Sigmund Freud Str. 25, 53127 Bonn, Germany

[3] Department of Biomedical Sciences, College of Veterinary Medicine, Cornell University, T4-018 Veterinary Research Tower, 14853-2703 Ithaca (NY), USA

[4] Department of Medicine, Heart and Vascular Institute and the McGowan Institute for Regenerative Medicine, University of Pittsburgh, School of Medicine, 3500 Terrace Street, S368 Scaife Hall, 15261 Pittsburgh (PA), USA

[5] Department of Pharmacology and Toxicology, Biomedical Center, University of Bonn, Sigmund Freud Str. 25, 53127 Bonn, Germany

Supplementary Materials:

Supplementary Figure 1. Cx43 expression in SkM, engraftment of SkM in infarcted hearts

Supplementary Figure 2. Proof of lentivirus injection into the scar, short term effects of Cx43 transduction on cardiac function.

Supplementary Figure 3. Long term Cx43 expression in the scar, effects on rhythm and function of the heart.

Supplementary Figure 4. Qualitative analysis of VT incidence after SkM engraftment and lvCx43/lvEGFP injection into the scar area.

Supplementary Figure 5. Voltage mapping signals from native and infarcted cardiac tissue.

Supplementary Figure 6. Original Western Blot data of EGFP and Cx43 expression in the myocardial scar area after direct intramyocardial injection of lentivirus constructs 2-3 days after infarction.

Supplementary Table 1. Basic ECG parameters.

Supplementary Video 1. Voltage mapping after lvEGFP transduction.

Supplementary Video 2. Voltage mapping after lvCx43 transduction.

Supplementary Figure 1 - Cx43 expression in SkM, engraftment of SkM in infarcted hearts

a) Surgical protocol for the SkM grafting experiments and their analysis.

b) Scheme of the dye transfer setup (left panel) using a glass micropipette. Dye loading is monitored via fluorescent microscopy (right panel).

c) Quantitative real time PCR analysis of scar areas of SkM engrafted hearts demonstrates expression of EGFP and of SkM-specific genes; expression levels are normalized with values obtained from non-SkM engrafted hearts.

d) Magnifications of the surface (left panel) and the intracardiac lead (right panel) ECG traces shown in Fig. 1g at the beginning of the stimulation train are shown. Capture (2:1) is proven by de-formed QRS-complexes (V) during stimulation (S). The dissociation of atrial (A) and ventricular (V) excitation during stimulation is clearly visible in the intracardiac atrial lead.

Supplementary Figure 2 - Proof of lentivirus injection into the scar, short term effects of Cx43 transduction on cardiac function.

a) PCR analysis against integrated lentiviral DNA in right ventricles (RV) and infarct areas (I.A.) of representative lvEGFP (n=3) and lvCx43 (n=2) injected hearts, respectively. Note positive signals in all transduced infarcts, but not in the RV.

b) Quantitative PCR demonstrates EGFP expression in the I.A. of lvEGFP (n=4) and lvCx43 (n=4) injected hearts, but not in in the native right (RV) and left (LV) ventricles of the same hearts, respectively.

c) Immunostainings against PECAM (red; native EGFP, green; nuclear Hoechst stain, blue) reveal that no endothelial cells are transduced. Scale bars: 10 µm.

d,e) Left ventricular catheterization 2 weeks after lentiviral gene transfer revealed similar stroke volume (d) and cardiac output (e) in lvEGFP and lvCx43 injected hearts.

Supplementary Figure 3 - Long term Cx43 expression in the scar, effects on rhythm and function of the heart.

a,b) Lack of Cx43 expression in the scar area two month after injection of lvEGFP (a) into the infarct (AEC staining, red), whereas Cx43 expression is detected after direct lvCx43 (b) gene transfer into the lesion; note the scattered islets of Cx43 expressing cells. Scale bars: 100µm.

c) Magnification of ECG traces from Fig. 4f reveals effective electrophysiological stimulation during burst stimulation in this VT protected heart upon lvCx43 injection: Ventricular capture (deformed QRS complexes in surface ECG during stimulation (S)) and atrio (A)-ventricular (V) dissociation (atrial lead, end of stimulation train) are shown.

d) Echocardiography demonstrates that left ventricular end-diastolic diameter (LVEDD) 8 weeks after gene transfer is similar in lvEGFP and lvCx43 treated mice.

Supplementary Figure 4 - Qualitative analysis of VT incidence after SkM engraftment and lvCx43/lvEGFP injection into the scar area.

In case of VT induction, in over 87% of animals (48/55) these lasted longer than 10 beats and/or could be evoked repetitively. Non self-limiting VT (n=3) were only observed in the SkM-EGFP group. The analysis demonstrates that Cx43-SkM engrafted and lvCx43 short- and long term injected hearts display a clearly lower degree of electrical vulnerability, because in the few remaining vulnerable hearts the more aggressive burst stimulation protocol is required for VT induction. The burst stimulation revealed the most striking differences in VT incidence between groups, differences were also noted for the extrastimulus protocol especially upon direct lvCx43 injection. In case VT were evoked by programmed stimulation, in over 82% (14/17) of these animals VT could be also induced by the burst stimulation protocol (levels of significance, *$p<0.05$, **$p<0.01$, ***$p<0.001$).

Supplementary Figure 5 - Voltage mapping signals from native and infarcted regions of the heart.

a,b) Images of the cardiac surface during voltage mapping experiments following lentiviral based gene transfer (left panels). Depicted voltage signals (right panels) were derived from native myocardium (black boxes and traces) and the encircled infarct area (red boxes and traces) of lvEGFP- (a) as well as lvCx43-hearts (b). Arrows indicate the site of electrical pacing.

c) Normalized (dF/F0) fluorescence signal amplitudes during voltage mapping of the infarct area (grey) and the native myocardium (black).

Supplementary Figure 6 – Original Western Blotting data of Cx43 expression in the myocardial scar area and left and right ventricles after I.A. injection of lvEGFP or lvCx43.

After injection of the control virus (lvEGFP, mouse No. 18 and 19) no Cx43 expression could be observed. In case of injection of the lvCx43 construct (mouse No. 4 and 5) into the I.A. 2-3 days after the initial surgery, expression of Cx43 could be detected 10-12 days after lentivirus application.

Group	HR [bpm]	P-wave [ms]	PQ-time [ms]	QRS-complex [ms]	QT-time [ms]
WT	422±69	13±3	40±6	17±3	40±2
SkM-EGFP	436±33	12±2	34,4±4 **	18±4	37±5
SkM-Cx43	418±70	12±2	54,6±13 **	20±3	41±5
lvEGFP	420±22	14±2	40±5	16±3	40±5
lvCx43	393±71	13±2	40±3	16±1	39±3
	n.s.	n.s.	p<0.01 SkM-Cx43 vs SkM-EGFP, others n.s.	n.s.	n.s.

Supplementary Table 1. Basic ECG parameters.

Prior to electrophysiological testing heart rate (HR), P-wave- and QRS-complex duration and PQ- and QT times were evaluated. Besides PQ-times in SkM-Cx43 mice vs EGFP-SkM, all the other parameters did not differ significantly between the groups.

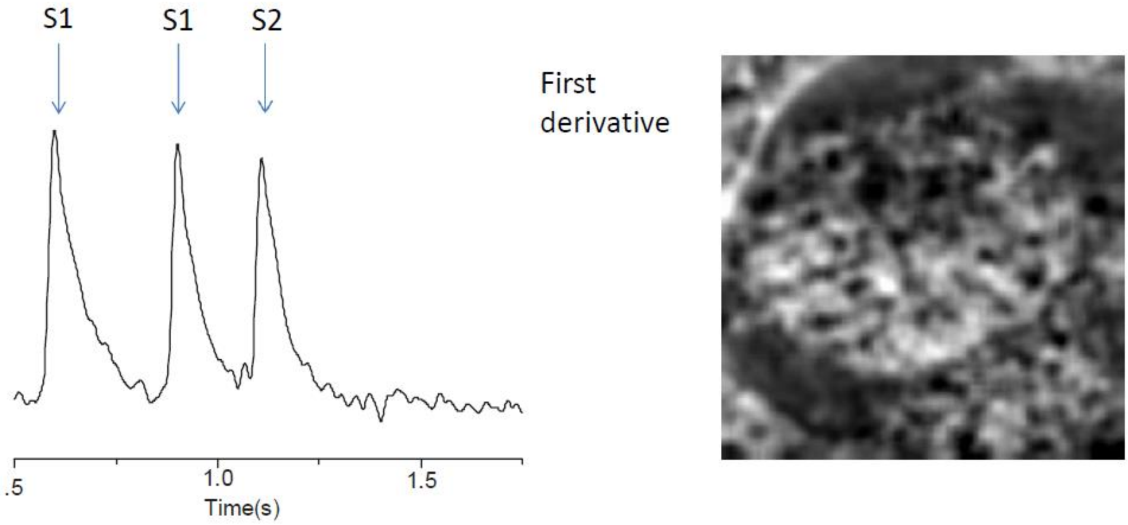

S1S2 pacing: 2 beats of S1 @ 300ms, S2 beat @ 200ms

Supplementary Video 1. Voltage mapping after lvEGFP transduction. Voltage mapping was performed 12 days postoperatively after transduction of the myocardial lesion with the lvEGFP construct using the membrane-potential-sensitive dye di-4-ANEPPS (l_{ex} 554±30 nm, l_{em} 640 nm). In the video the heart is stimulated from the base of the heart at 300 ms intervals. The lesion is as far as possible electrically isolated from the surrounding native myocardium, since the wavefront orbits the lesion. By a subsequent 200 ms extrastimulus, spiral waves as a VT correlate are provoked.

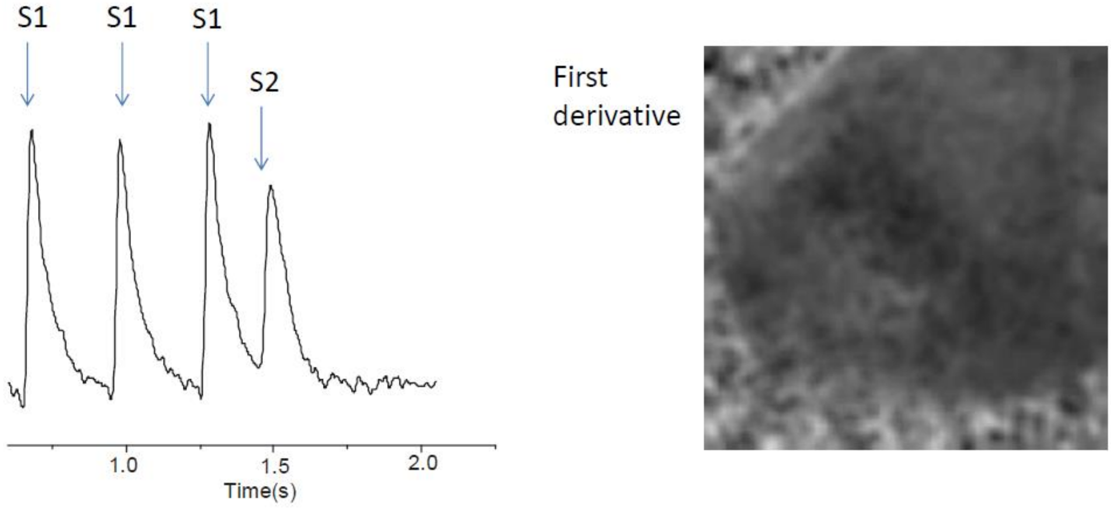

S1S2 Pacing. 3beats of S1 @ 300ms, S2 beat @ 200ms

Supplementary Video 2. **Voltage mapping after lvCx43 transduction**. In contrast to supplementary video 1 following transduction of the myocardial lesion with lvCx43 the electrical wavefront partially enters the infarct zone, the remaining electrically isolated area is much smaller and by the extrastimulus protocol no ventricular arrhythmia can be induced.

Roell et al., Suppl. Figure 1

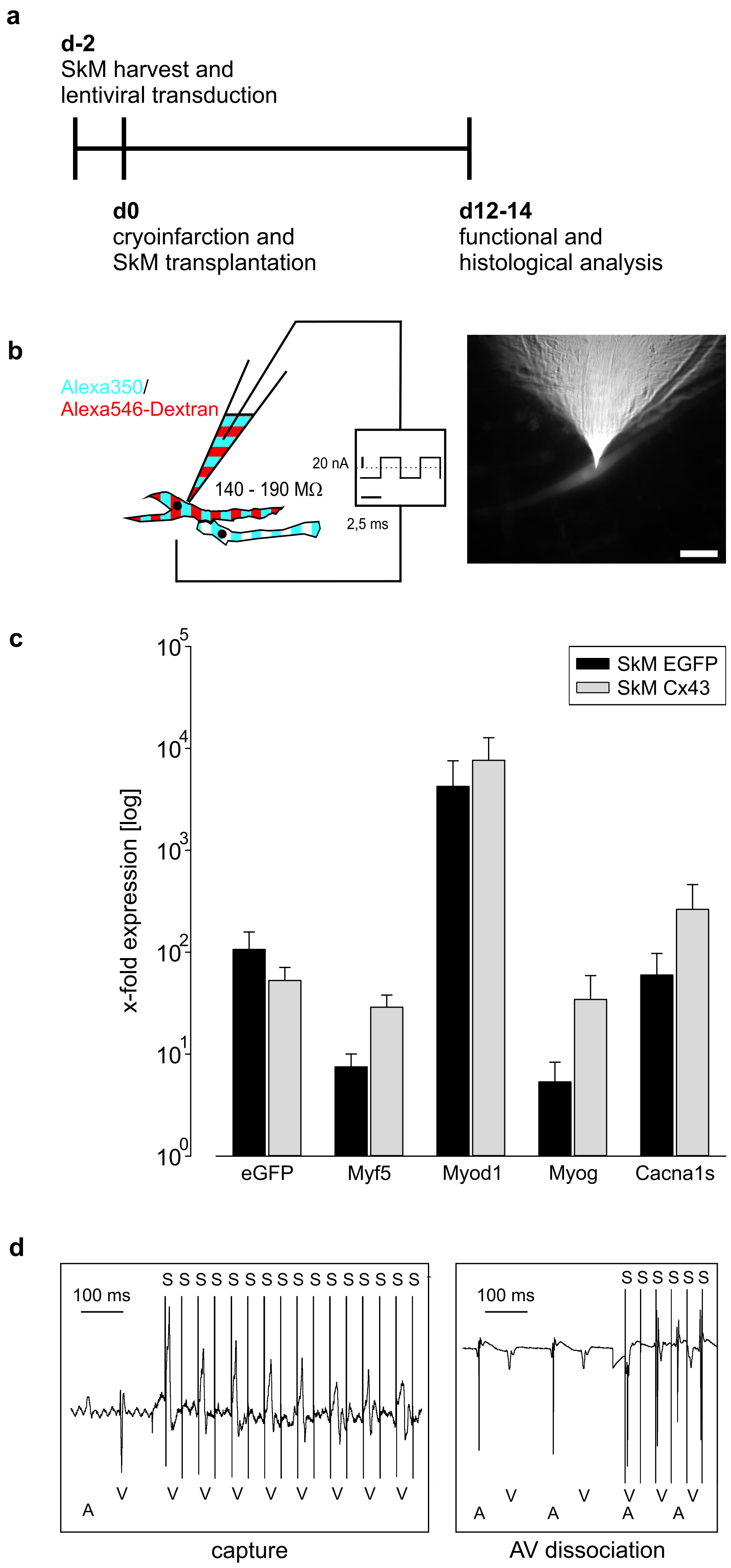

Roell et al., Suppl. Figure 2

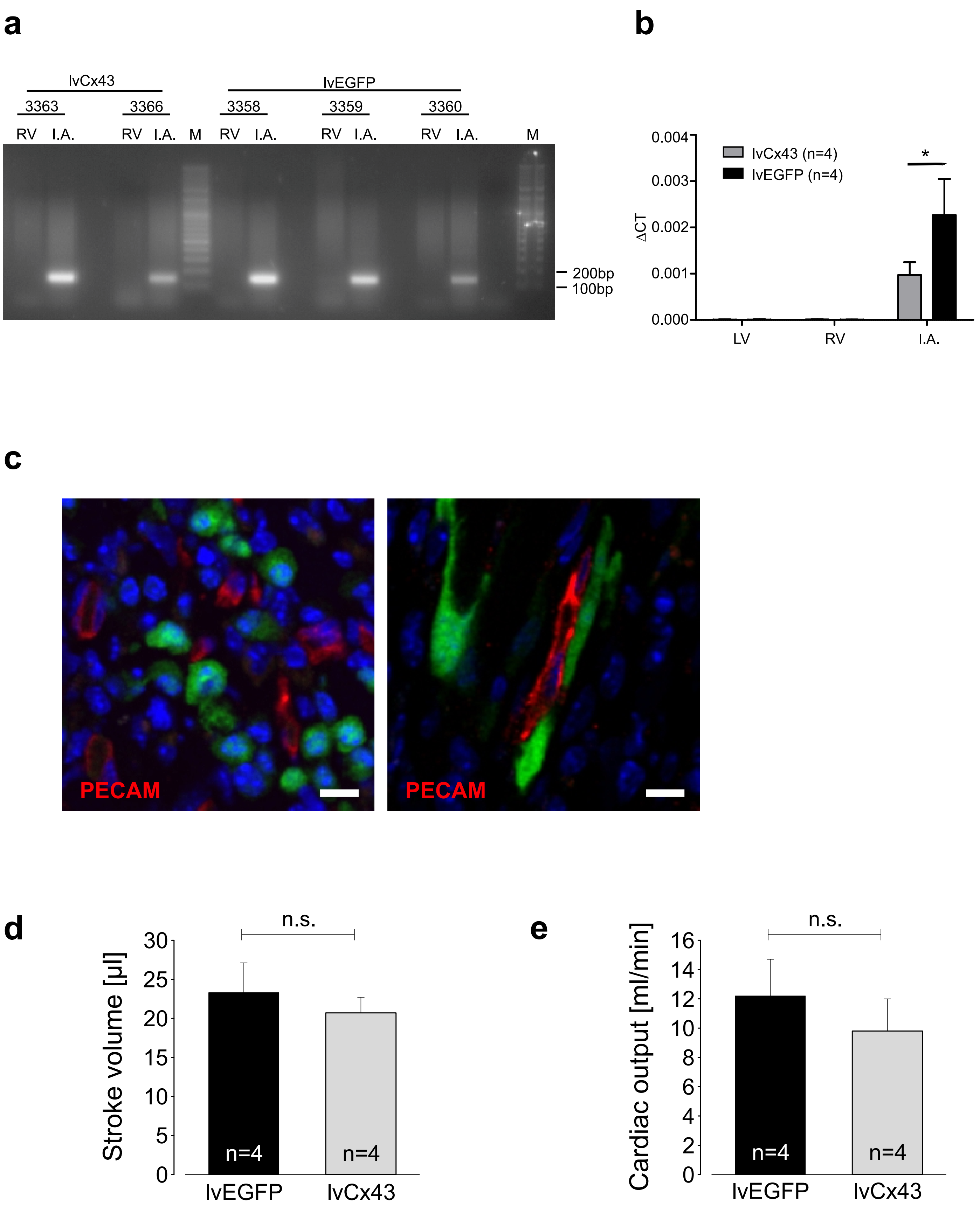

Roell et al., Suppl. Figure 3

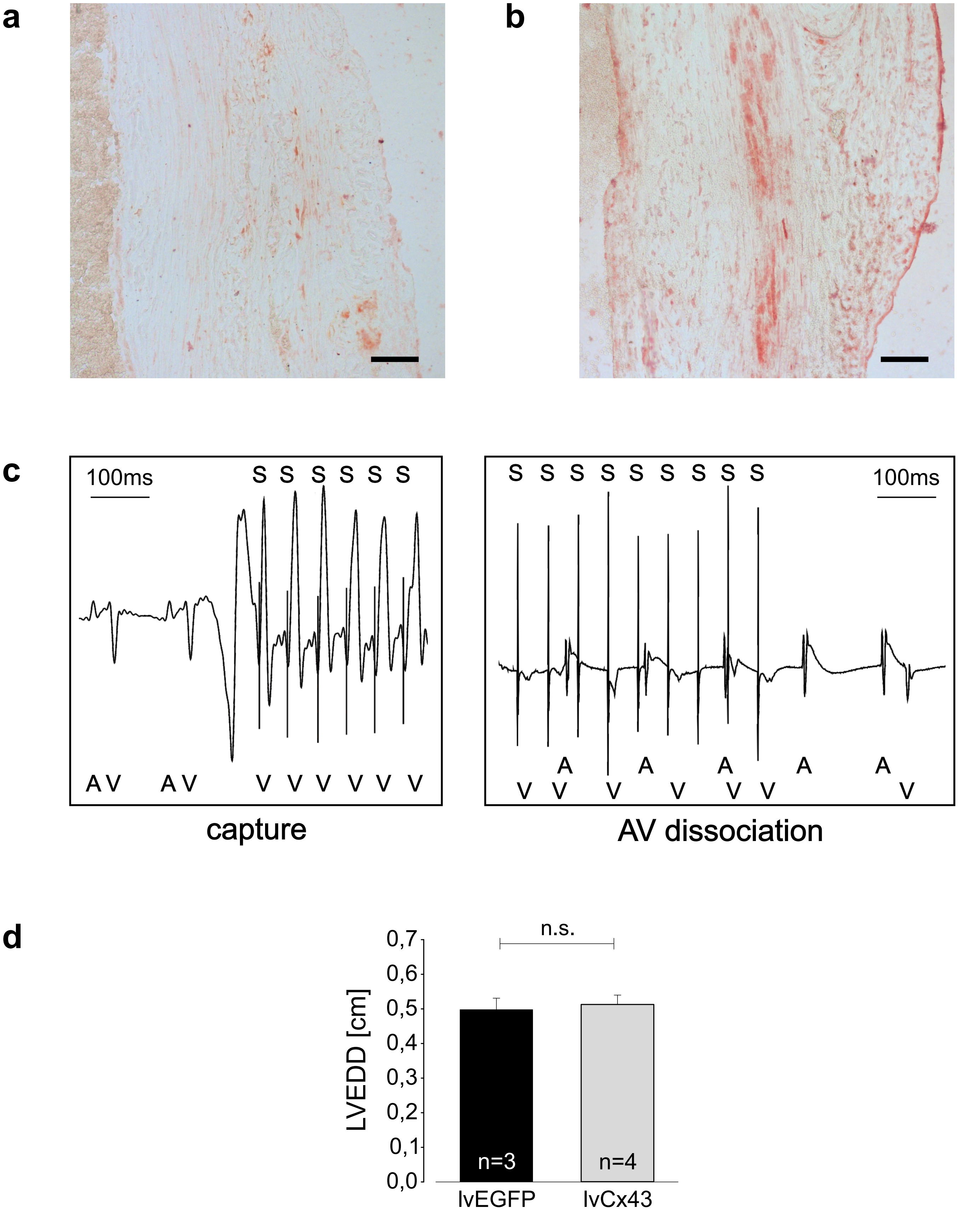

Roell et al., Suppl. Figure 4

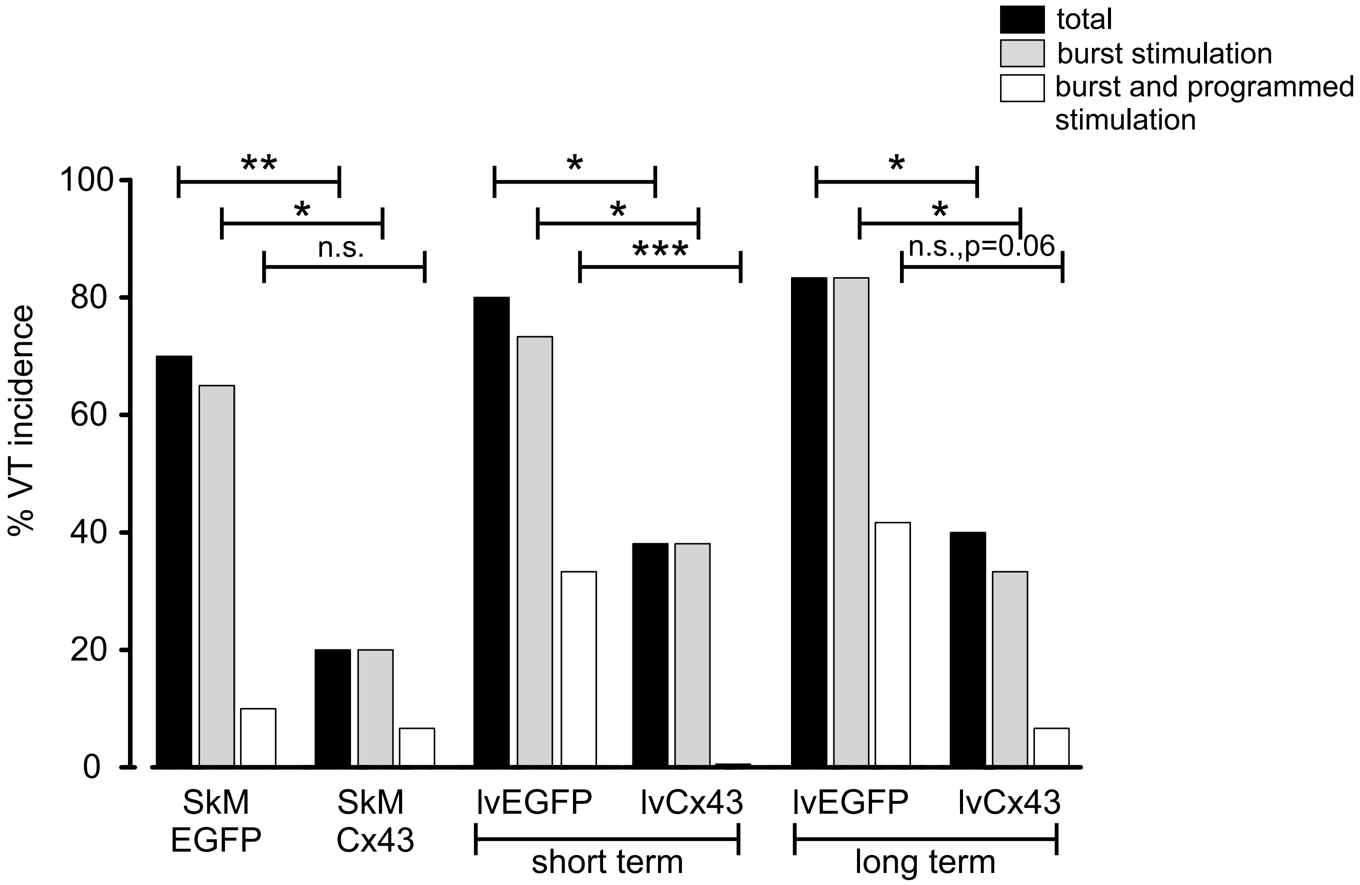

Roell et al., Suppl. Figure 5

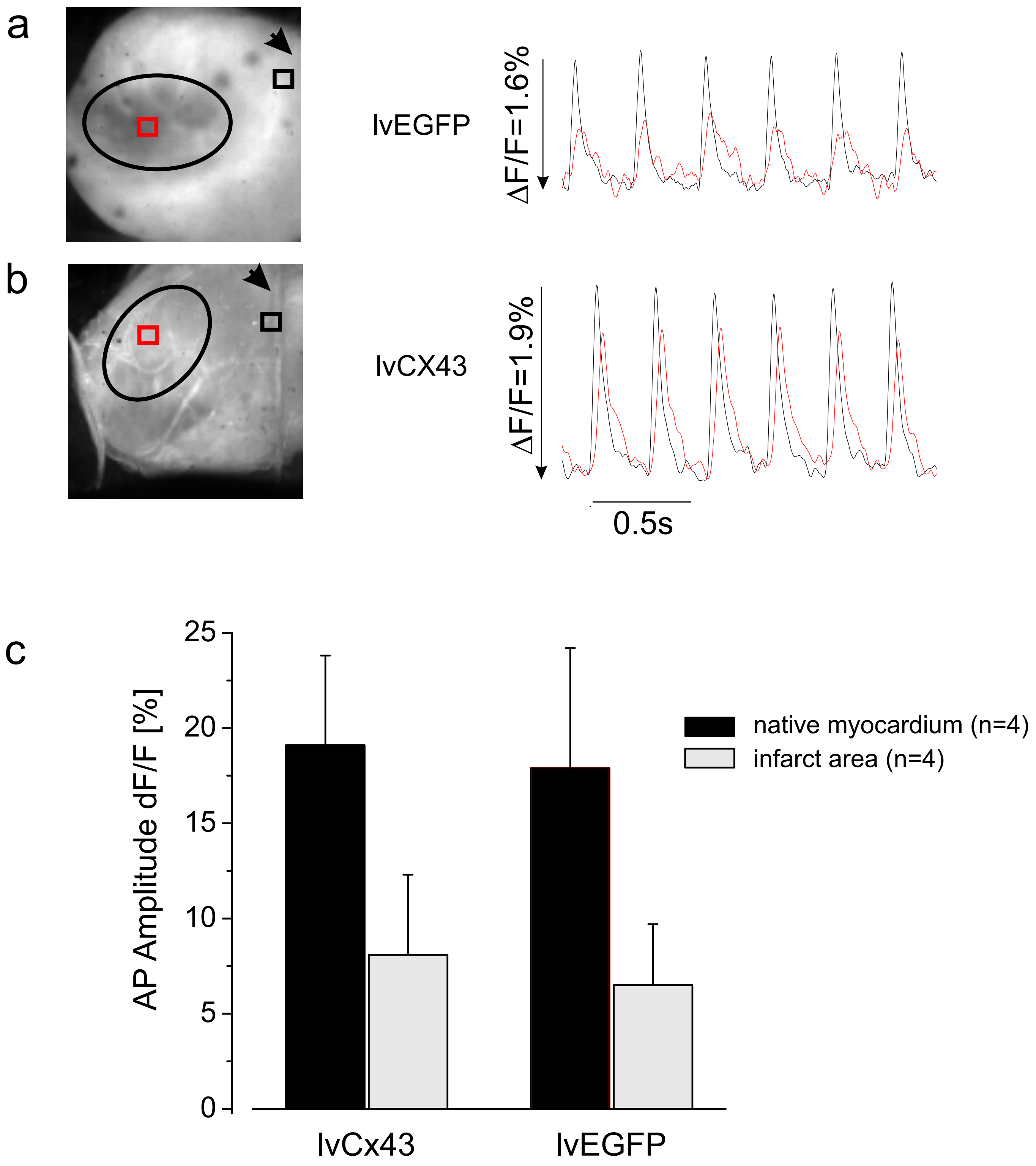

Roell et al., Suppl. Figure 6

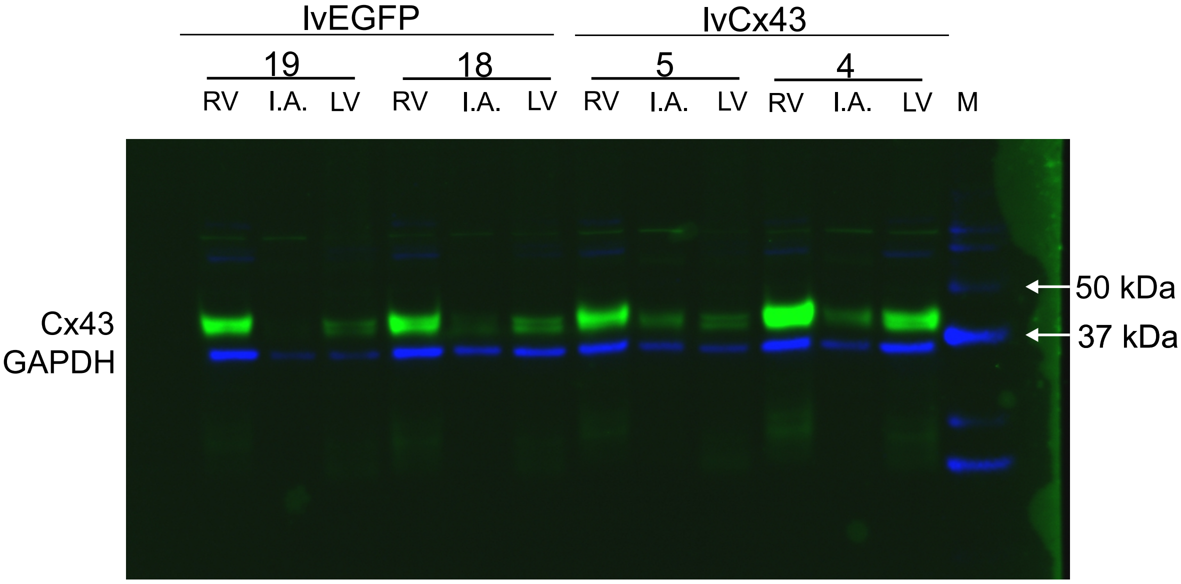

Supplement: Supplementary file 1 — Supplementary Materials [file 41598_2018_25147_MOESM1_ESM.pdf]
